# EPS8L2 drives colorectal cancer cell proliferation and migration via YBX1-dependent activation of G3BP2 transcription

**DOI:** 10.1038/s41419-025-07929-x

**Published:** 2025-08-10

**Authors:** Yimeng Duan, Peixian Li, Yanmei Yang, Guanghua Wu, Hao Xing, Hong Chen, Liangbo Zhao, Lei Liu, Xiao Sun, Shuiling Jin, Luyun He, Benyu Liu

**Affiliations:** 1 State Key Laboratory of Metabolic Dysregulation & Prevention and Treatment of Esophageal Cancer, Tianjian Laboratory of Advanced Biomedical Sciences, Academy of Medical Sciences, Zhengzhou University, Zhengzhou, China; 2 Department of Pathophysiology, School of Basic Medical Sciences, Zhengzhou University, Zhengzhou, China; 3 Department of Oncology, the First Affiliated Hospital of Zhengzhou University, Zhengzhou, China

**Keywords:** Oncogenes, Mechanisms of disease

## Abstract

Colorectal cancer (CRC) remains a leading cause of cancer-related mortality worldwide, characterized by molecular heterogeneity and limited therapeutic options. Here, we identified EPS8L2 as a novel driver of colorectal tumorigenesis. EPS8L2 is significantly upregulated in CRC tissues and negatively correlated with patients’ prognosis. Functionally, upregulation of EPS8L2 promotes proliferation and metastasis of CRC cells in vitro and in vivo, and vice versa. Similarly, EPS8L2 overexpression promotes patient-derived organoids growth. Mechanistically, EPS8L2 increases YBX1 phosphorylation by enhancing its interaction with phosphokinase S6K1. Phosphorylated YBX1 translocates into nucleus and initiates G3BP2 transcription, leading to activation of the MAPK signaling pathway. Moreover, knockout of *Eps8l2* impairs CRC tumorigenesis in the AOM/DSS induced mouse model. In summary, we revealed a novel EPS8L2-YBX1-G3BP2 regulatory axis involved in CRC progression, which provides a new theoretical basis for tumor therapy.

## Introduction

Colorectal cancer (CRC), including both colon and rectal cancers, ranks as the second leading cause of cancer-related deaths [1]. Its pathogenesis typically initiates cumulative genetic and epigenetic alterations in colonic epithelium, driving aberrant cellular proliferation and malignant transformation [2]. While early detection programs have improved 5-year survival rates, ~25% of patients present with stage IV disease at diagnosis, and an additional 25–50% of initially early-stage patients progress to metastatic CRC [3]. The prognosis of metastatic CRC patients remains poor, with a median 5-year survival rate of only 12.5% [4]. Therapeutic strategies diverge significantly by disease stage. Surgical resection is the preferred treatment option for early-stage CRC, while advanced or recurrent CRC typically requires multidisciplinary approach, combining chemotherapy, targeted therapy, and immunotherapy [5]. Despite therapeutic advances, metastasis and recurrence persist as principal barriers to survival improvement. Consequently, deciphering the molecular drivers of CRC progression and identifying specific biomarkers are critical imperatives for enhancing the prognosis of CRC patients.

Receptor tyrosine kinases (RTKs) are critical transmembrane receptors responsible for transmitting extracellular signals into intracellular [6]. They regulate essential processes like cell growth, differentiation, and survival [7], making them important targets for cancer therapy [8]. Among RTK family, the epidermal growth factor receptor (EGFR) is particularly well-studied in cancer. When activated by ligands (e.g., EGF), EGFR triggers key pathways including RAS-RAF-MAPK and PI3K-AKT, which drive cancer cell proliferation and metastasis [9, 10]. Therefore, exploring and understanding the functions and mechanisms of RTK-related genes remains crucial for developing better CRC treatments and understanding disease mechanisms.

EPS8, a conserved intracellular substrate of the EGFR pathway, is widely expressed across species, including humans, nematodes, and fruit flies [11]. The EPS8 protein family comprises four structurally homologous members (EPS8, EPS8L1, EPS8L2, and EPS8L3), all sharing key functional domains: an N-terminal phosphotyrosine-binding (PTB) domain, central SH3 domain, and C-terminal effector region [12]. Members of the EPS8 family have been widely reported to be closely associated with various cancers [13–15]. Current studies about EPS8L2 primarily focused on auditory system [16–18], where its loss causes hearing impairment [16, 17]. EPS8L2 has also been reported to be involved in cytoskeletal reorganization, urinary extracellular vesicle production, and nuclear movement during cell migration [19–21]. Moreover, EPS8L2 is associated with T cell activation, lupus nephritis, and diabetic erectile dysfunction [22–24]. In cancer, EPS8L2 was reported to be associated with testicular germ cell tumors’ drug resistance [25]. Additionally, high expression of EPS8L2 is a biomarker of endometrial carcinoma, renal cell carcinoma, and rhabdomyosarcoma [26–28]. However, the function and mechanism of EPS8L2 in CRC development remain unknown.

Here, we identified EPS8L2 was upregulated in CRC through bioinformatics analysis and CRC tissue microarray. We found that elevated EPS8L2 expression was significantly associated with tumor grade, lymph node metastasis, and CRC patients’ poor prognosis. Additionally, we showed that EPS8L2 promoted CRC cell proliferation and metastasis by facilitating S6K1-mediated YBX1 phosphorylation and subsequent nuclear translocation to activate G3BP2 transcription and the MAPK signaling pathway. Furthermore, *Eps8l2* knockout led to decreased tumor incidence in an AOM/DSS-induced carcinogenesis model. Altogether, this study elucidated a previously unknown biological function and mechanism of EPS8L2 in CRC, which may provide a promising treatment strategy for CRC intervention.

## Results

### EPS8L2 is upregulated in CRC

**Fig. 1:**
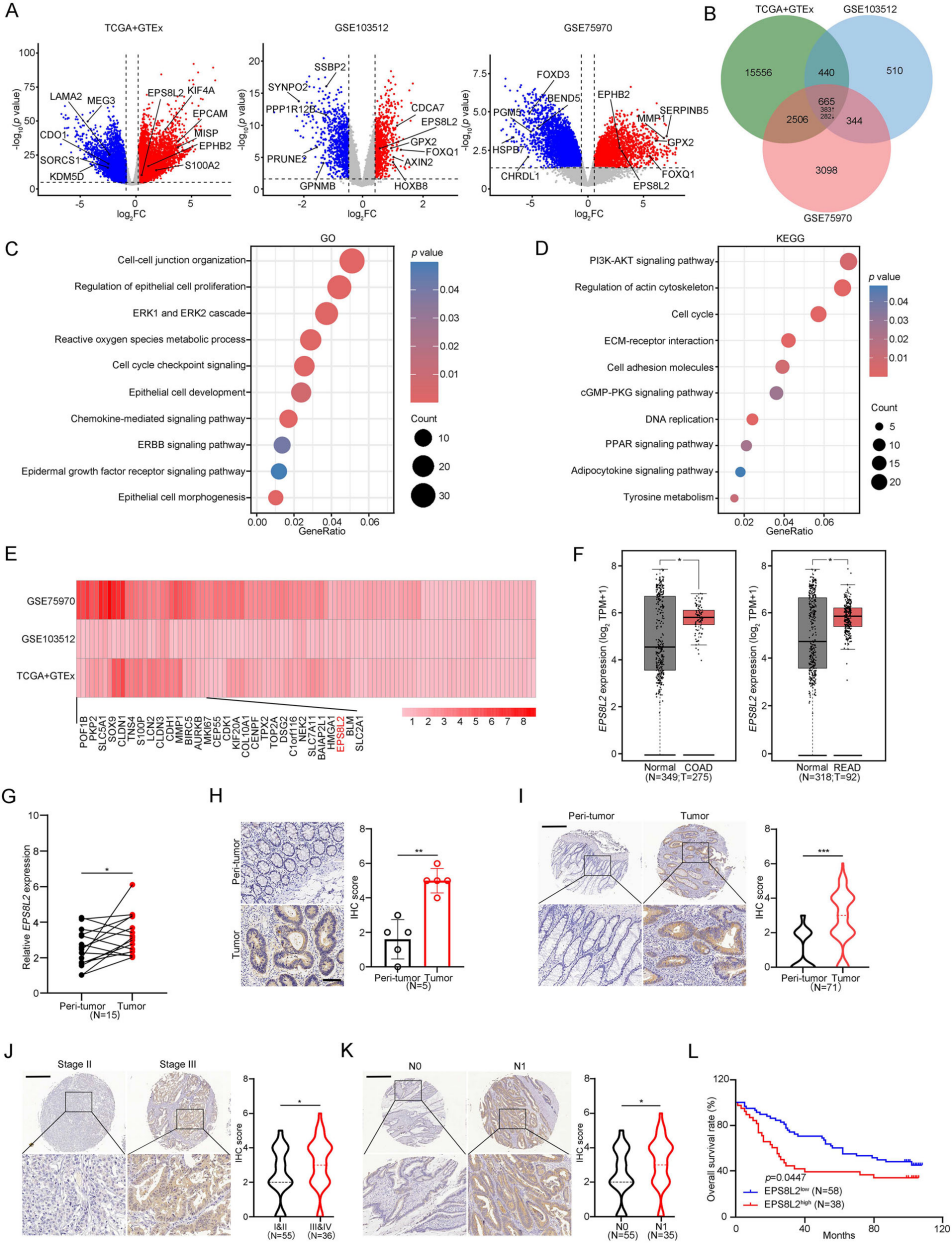
EPS8L2 is upregulated in CRC. **A**, **B** DEGs in TCGA+GTEx, GSE103512, and GSE75970 datasets were illustrated using volcano plots (**A**) and venn diagram (**B**). **C, D** GO (**C**) and KEGG (**D**) enrichment analysis of 665 common DEGs in (**B**). **E** 104 upregulated RTK related genes among three datasets were displayed by heatmap. **F** EPS8L2 expression in COAD and READ according to GEPIA website. COAD: colon adenocarcinoma, READ: rectal adenocarcinoma. Boxplot lower boundary represents the first quartile, the upper boundary represents the third quartile, and the median is indicated by solid horizontal line. **G** EPS8L2 expression in 15 pairs of CRC samples was examined by RT-qPCR. **H** EPS8L2 expression was detected by IHC staining in 5 pairs of CRC samples. Scale bars, 100 μm. Representative images were shown in left panel, and statistical results of IHC score were shown in right panel. **I**–**K** EPS8L2 expression was detected by IHC staining in CRC tissue array. Representative images of EPS8L2 expression in paired tissues (**I**), different pathological grading tissues (**J**), and lymph node metastasis tissues (**K**) were shown in left panel. The correspondingly statistical results of IHC score were shown in right panel by violin plots (**I**–**K**). The dashed lines indicate the median IHC score for each group. N0 represents patients without lymph node metastasis, and N1 represents patients with localized lymph node metastasis in (**K**). Scale bars, 500 μm. **L** Relationship between EPS8L2 expression and CRC patients’ overall survival according to CRC tissue array. Data are presented as means ± SD. ***p < 0.001, **p < 0.01, *p < 0.05.

To explore potential regulators involved in CRC development, we compared gene expression profiles in several publicly accessible human CRC datasets from the Cancer Genome Atlas (TCGA) and the Gene Expression Omnibus (GEO) databases. We identified 665 overlapping differentially expressed genes (DEGs) based on a significant threshold of |log_2_FC| ≥ 0.58 and *p* < 0.05 (Fig. 1A, B). We then performed Gene Ontology (GO) and Kyoto Encyclopedia of Genes and Genomes (KEGG) enrichment analysis using the 665 common DEGs and found that the RTK signaling was significantly enriched, such as ERK1 and ERK2 cascade, and PI3K-AKT signaling pathway (Fig. 1C, D). Therefore, we focused on RTK-related genes and analyzed their expression changes in CRC tissues compared to normal controls (Fig. 1E and Supplementary Fig. 1A). The thirty most upregulated genes in CRC tissues were analyzed, and only EPS8L2’s function in CRC remains unclear. We therefore selected EPS8L2 for following exploration. The upregulation of EPS8L2 mRNA and protein levels in CRC was further validated (Fig. 1F–H). We then obtained a human CRC tissue microarray (TMA) and verified that EPS8L2 was highly expressed in tumor tissues compared with paired peri-tumor tissues (Fig. 1I). In addition, EPS8L2 expression level was increased in CRC samples with III and IV clinical stages (Fig. 1J) or lymph node metastasis (Fig. 1K and Supplementary Fig. 1B). Moreover, high expression of EPS8L2 was associated with poor prognosis (Fig. 1L and Supplementary Fig. 1C). Conclusively, EPS8L2 is highly expressed in CRC and positively correlated with poor prognosis.

### EPS8L2 promotes CRC cell proliferation and migration

**Fig. 2:**
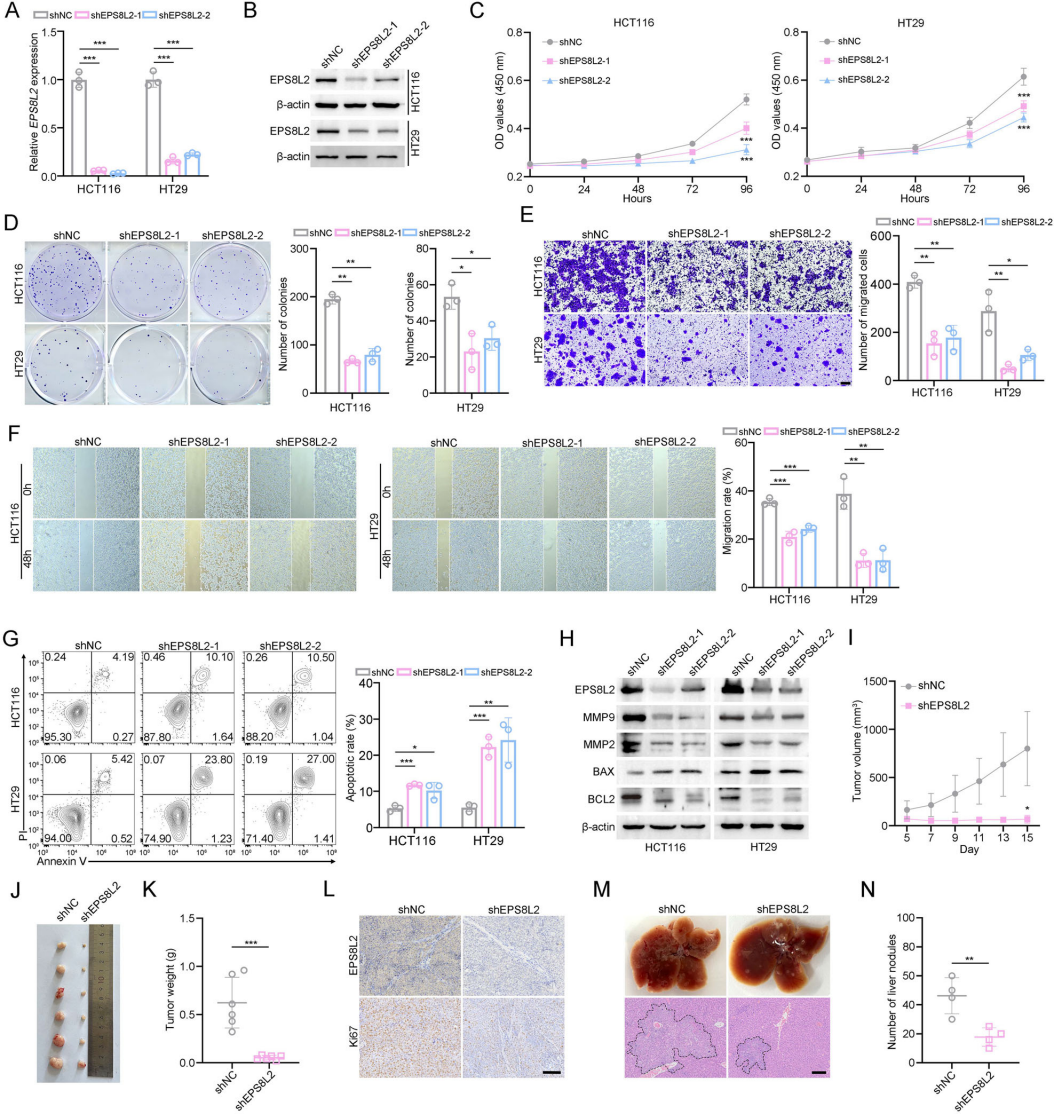
EPS8L2 knockdown inhibits CRC cell proliferation and migration. **A**, **B** EPS8L2 expression were examined by RT-qPCR (**A**) and western blotting (**B**) in EPS8L2 knockdown HCT116 and HT29 cells. **C, D** Cell proliferation was measured using CCK-8 assay (**C**) and colony formation assay (**D**) after EPS8L2 knockdown in HCT116 and HT29 cells. **E**, **F** Transwell migration (**E**) and wound healing (**F**) assays were conducted to assess the migratory ability of HCT116 and HT29 cells after EPS8L2 knockdown. Scale bars, 100 μm. **G** FACS measured HCT116 and HT29 cell apoptotic rate after EPS8L2 knockdown. Representative images were shown in left panel and statistical results were shown in right panel. **H** Expression of EPS8L2, MMP9, MMP2, BAX, and BCL2 was detected by western blotting after EPS8L2 knockdown in HCT116 and HT29 cells. **I**–**K** EPS8L2 knockdown and control HCT116 cells were injected into BALB/c nude mice for 15 days (*n* = 6 per group). Statistical results of tumor volumes (**I**), images of subcutaneous xenograft tumors (**J**), and statistical results of tumor weights (**K**) were shown. **L** Expression of EPS8L2 and Ki67 in subcutaneous xenograft tumors was detected by IHC staining. Scale bars, 100 μm. **M, N** EPS8L2 knockdown and control HCT116 cells were injected into tail vein of BALB/c nude mice for 33 days (*n* = 4 per group). Representative images of liver metastasis nodules and HE staining (**M**) and quantification of liver metastatic nodules were shown (**N**). Scale bars, 500 μm. Data are presented as means ± SD. ***p < 0.001, **p < 0.01, *p < 0.05.

To investigate its function, stable EPS8L2 knockdown cell lines were successfully established (Fig. 2A, B). We found that EPS8L2 silencing markedly inhibited cell proliferation as shown by CCK-8 assay and colony formation assay (Fig. 2C, D). Furthermore, transwell migration and wound healing assays were conducted and revealed that EPS8L2 knockdown reduced the migratory ability of HCT116 and HT29 cells (Fig. 2E, F). Interestingly, knockdown of EPS8L2 resulted in increased apoptosis (Fig. 2G), which was further validated by enhanced BAX expression and downregulation of BCL2 (Fig. 2H). To explore its role in vivo, we subcutaneously implanted EPS8L2-depleted HCT116 cells into BALB/c nude mice. The results showed that EPS8L2 knockdown significantly decreased tumor volume and weight in nude mice (Fig. 2I–K). Consistently, Ki67 expression was obviously inhibited after EPS8L2 knockdown (Fig. 2L). We then performed an in vivo metastasis assay by injecting EPS8L2 knockdown (shEPS8L2) or control (shNC) HCT116 cells into the tail vein of BALB/c nude mice. We observed a significant reduction in liver metastatic nodules in shEPS8L2 group (Fig. 2M, N).

**Fig. 3:**
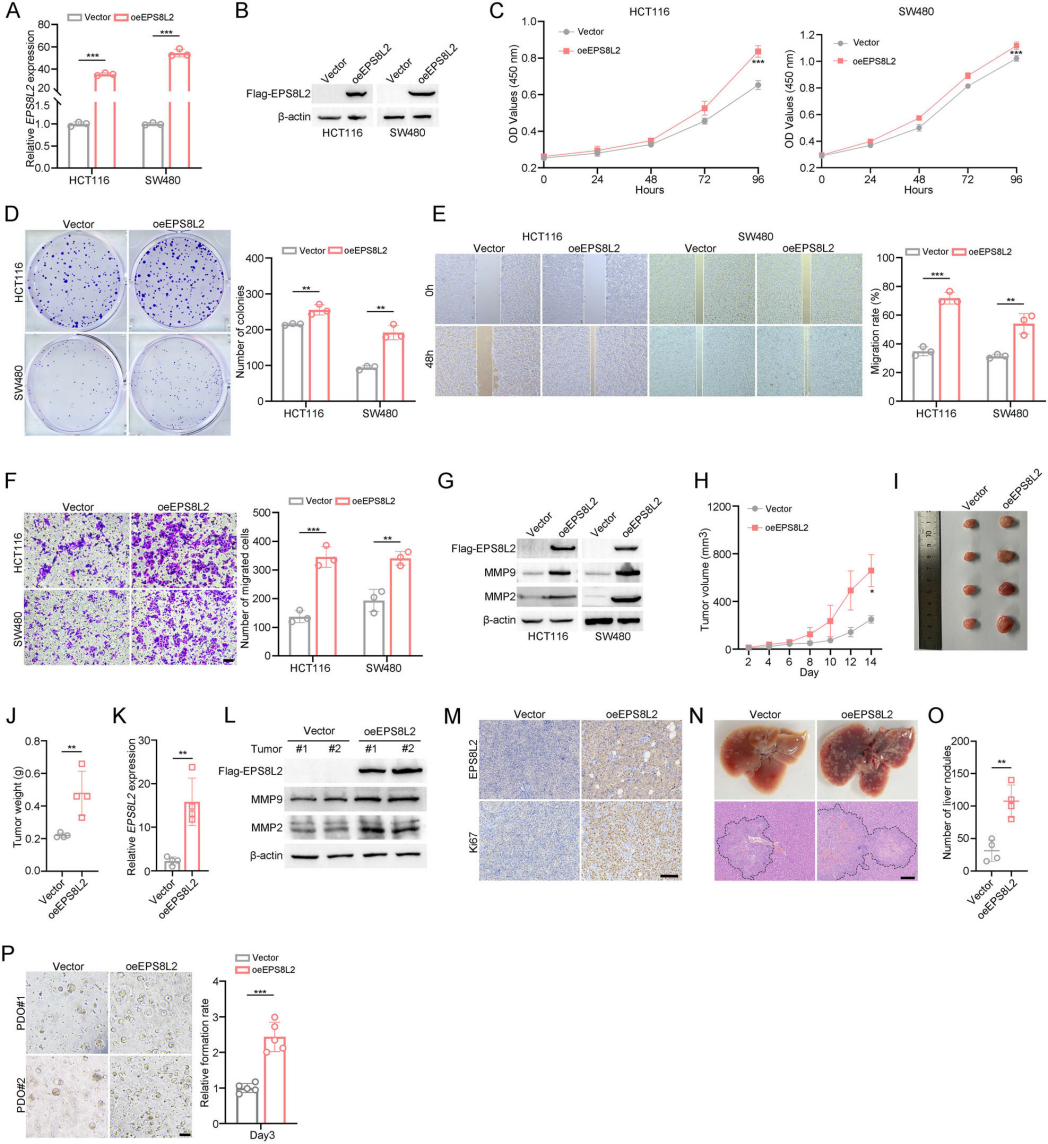
EPS8L2 overexpression promotes CRC cell growth and invasion. **A**, **B** EPS8L2 expression were examined by RT-qPCR (**A**) and western blotting (**B**) in EPS8L2 overexpression HCT116 and SW480 cells. **C**, **D** Cell proliferation was measured using CCK-8 assay (**C**) and colony formation assay (**D**) after EPS8L2 overexpression in HCT116 and SW480 cells. **E**, **F** Wound healing (**E**) and transwell migration (**F**) assays were conducted to assess the migratory ability of HCT116 and SW480 cells after EPS8L2 overexpression. Scale bars, 100 μm. Representative images were shown in left panel and statistical results were shown in right panel. **G** Expression of EPS8L2, MMP9, MMP2, BAX, and BCL2 was detected by western blotting after EPS8L2 overexpression in HCT116 and SW480 cells. **H**–**J** EPS8L2 overexpression and vector HCT116 cells were injected into BALB/c nude mice for 14 days (*n* = 4 per group). Statistical results of tumor volumes (**H**), images of subcutaneous xenograft tumors (**I**), and statistical results of tumor weights (**J**) were shown. **K** Expression of EPS8L2 was detected by RT-qPCR in vector and oeEPS8L2 group of subcutaneous xenograft tumors. **L** Expression of EPS8L2, MMP9, MMP2 was detected by western blotting in vector and oeEPS8L2 group of subcutaneous xenograft tumors. **M** Expression of EPS8L2 and Ki67 in subcutaneous xenograft tumors were detected by IHC staining. Scale bars, 100 μm. **N**, **O** EPS8L2 overexpression and vector HCT116 cells were injected into tail vein of BALB/c nude mice for 28 days (*n* = 4 per group). Representative images of liver metastasis nodules and HE staining (**N**) and quantification of the liver metastatic nodules were shown (**O**). Scale bars, 500 μm. **P** Representative images of patient-derived organoids (PDOs) images at day 3 are shown in left panel, and quantitative analysis of relative formation rate is shown in right panel. Scale bars, 100 μm. Data are presented as means ± SD. ***p < 0.001, **p < 0.01, *p < 0.05.

To further confirm above findings, we generated EPS8L2-overexpressing HCT116 and SW480 cell lines (Fig. 3A, B). We found that EPS8L2 overexpression greatly enhanced the proliferation and migration capacities of HCT116 and SW480 cells in vitro (Fig. 3C–F). Importantly, the expression levels of epithelial-mesenchymal transition (EMT) markers, MMP2 and MMP9, were also significantly upregulated after EPS8L2 overexpression (Fig. 3G). We also overexpressed EPS8L2 in RKO cells, which lacks endogenous EPS8L2 expression (Supplementary Fig. 2A, B). Consistently, EPS8L2 ectopic expression promoted RKO cell proliferation and migration in vitro (Supplementary Fig. 2C–E). Next, xenograft and in vivo metastasis assays were carried out using EPS8L2 overexpressing HCT116 cells. The results indicated that EPS8L2 overexpression increased tumor volume and weight (Fig. 3H–J). The upregulation of EPS8L2 in tumor tissues was validated by RT-qPCR, western blotting, and IHC (Fig. 3K–M). Besides, MMP9, MMP2, and Ki67 expression levels were increased after EPS8L2 overexpression (Fig. 3L, M). Similarly, we observed a significant increase in liver metastatic nodules in the oeEPS8L2 group after injecting HCT116 cells into the tail vein (Fig. 3N, O). To further assess its preclinical relevance, we obtained fresh CRC samples and conducted patient-derived organoids (PDOs) experiments. We found that EPS8L2 overexpression promoted organoids growth (Fig. 3P). Collectively, EPS8L2 promotes CRC cell proliferation and migration in vitro and in vivo.

### EPS8L2 activates the MAPK signaling pathway

**Fig. 4:**
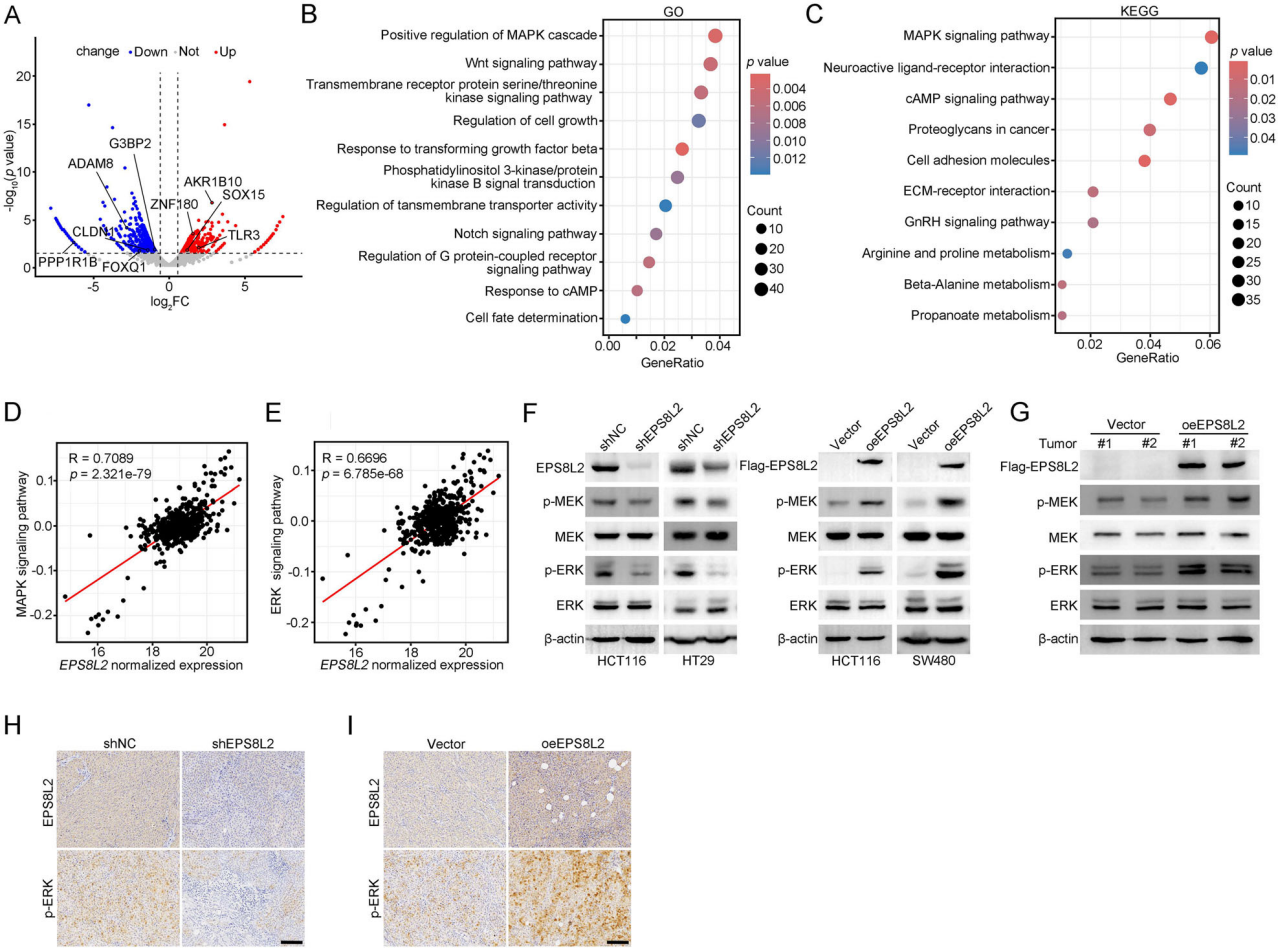
EPS8L2 activates MAPK signaling pathway. **A** DEGs between shEPS8L2 and shNC HCT116 cells were shown by volcano plot. DEGs are defined by *p* value < 0.05 and Log_2_|Fold Change| ≥ 0.56. **B**, **C** GO (**B**) and KEGG (**C**) enrichment analysis of DEGs in (**A**). **D**, **E** Correlation analysis between EPS8L2 expression and MAPK signaling pathway (**D**) or ERK signaling pathway (**E**) according to TCGA database. **F** Expression of EPS8L2, p-MEK, MEK, p-ERK, and ERK was detected by western blotting in EPS8L2 knockdown and overexpression CRC cells. **G** Expression of EPS8L2, p-MEK, MEK, p-ERK, and ERK was detected by western blotting in vector and oeEPS8L2 groups of subcutaneous xenograft tumors. **H**, **I** Expression of EPS8L2 and p-ERK was detected by IHC staining in EPS8L2 knockdown (**H**) and overexpression (**I**) subcutaneous xenograft tumors. Scale bars, 100 μm.

To determine the underlying mechanism, RNA sequencing was performed using EPS8L2 knockdown HCT116 cells. 1430 DEGs were identified in shEPS8L2 cells compared to shNC groups, including 534 upregulated genes and 896 downregulated genes (Fig. 4A). Notably, several reported oncogenes were downregulated after EPS8L2 silencing, such as FOXQ1, G3BP2, ADAM8, CLDN1, and PPP1R1B [29–33]. GO and KEGG analyses revealed that these DEGs were predominantly enriched in the MAPK signaling pathway (Fig. 4B, C). Correlation analysis using TCGA database also revealed significant positive correlation between EPS8L2 level and activation of the MAPK or ERK signaling pathway (Fig. 4D, E), suggesting that EPS8L2 may be involved in the activation of the MAPK signaling. To confirm it, we performed western blotting and IHC assays, and found that EPS8L2 knockdown inhibited the phosphorylation of ERK and MEK in CRC cell lines and tissues, and vice versa (Fig. 4F–I). Collectively, EPS8L2 activates the MAPK signaling pathway.

### EPS8L2 facilitates YBX1 nuclear accumulation by enhancing its phosphorylation

**Fig. 5:**
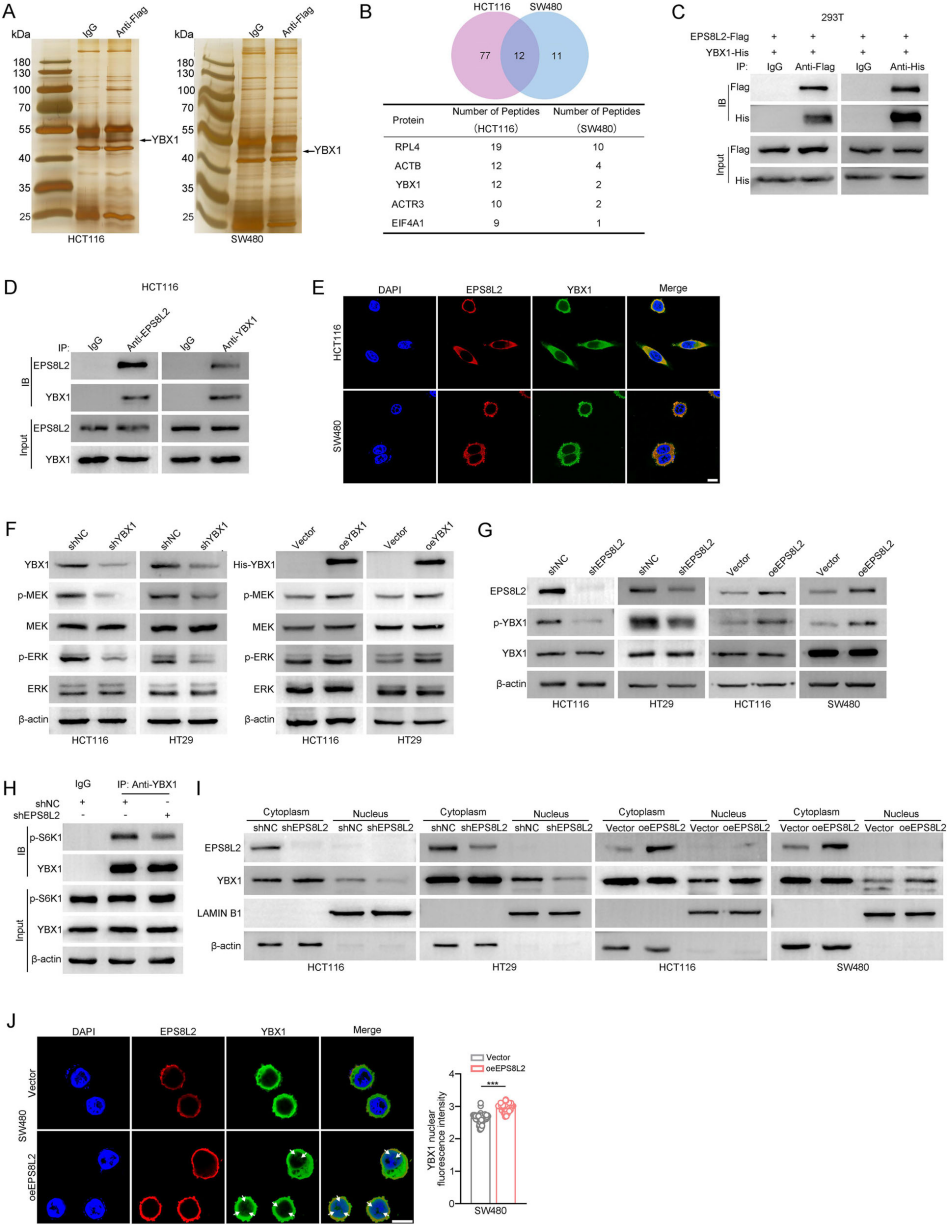
EPS8L2 facilitates YBX1 translocation into nucleus by enhancing its phosphorylation. **A** The extracted protein was precipitated with anti-Flag antibody and separated by SDS-PAGE followed by silver staining in HCT116 and SW480 cells. **B** Common proteins identified by mass spectrometry in HCT116 and SW480 cells that interact with EPS8L2 were shown by venn diagram. The top five interacting proteins are listed below. **C** The interaction between exogenous EPS8L2 and exogenous YBX1 was assessed in HEK293T cells. **D** The interaction between endogenous EPS8L2 and endogenous YBX1 was assessed in HCT116 cells. **E** Immunofluorescence staining of EPS8L2 and YBX1 in HCT116 and SW480 cells. Scale bar, 10 μm. **F** Expression of YBX1, p-MEK, MEK, p-ERK, and ERK in YBX1 knockdown and overexpression CRC cells was detected by western blotting. **G** Expression of p-YBX1 and total YBX1 was determined by western blotting in EPS8L2 knockdown and overexpression CRC cells. **H** Co-IP assays were performed to examine the interaction between YBX1 and p-S6K1. **I** Expression of YBX1 after nucleoplasm separation was detected by western blotting in EPS8L2 knockdown and overexpression CRC cells. **J** Immunofluorescence to detected the change of nuclear YBX1 in SW480 cells with EPS8L2 changes. Representative images were shown in left panel and statistical results were shown in right panel. Scale bars, 10 μm. Data are presented as means ± SD. ***p < 0.001.

To further elucidate how EPS8L2 activates MAPK signaling pathway, we performed immunoprecipitation-mass spectrometry (IP-MS) assay to screen potential EPS8L2-interacting proteins. The silver staining results showed a distinct differential band between 40 kDa and 55 kDa in the Flag antibody groups compared to the IgG control groups (Fig. 5A). This differential band was then subjected to mass spectrometry analysis, which identified 89 interacting proteins in HCT116 cells and 23 interacting proteins in SW480 cells (Fig. 5B). YBX1 scored high and was selected for validation (Fig. 5B and Supplementary Fig. 3A, B). The interaction between EPS8L2 and YBX1 was confirmed by co-immunoprecipitation (co-IP) and immunofluorescence (IF) assays (Fig. 5C–E). In addition, we examined effect of YBX1 on MAPK signaling pathway. The results showed that YBX1 knockdown significantly reduced the levels of p-MEK and p-ERK in both HCT116 and HT29 cells, and vice versa (Fig. 5F). Interestingly, it was observed that YBX1 was also highly expressed in CRC (Supplementary Fig. 3C).

YBX1 is primarily localized in the cytoplasm [34, 35]. However, in response to cellular stress, such as ultraviolet (UV) radiation, heat shock, or oxidative stress, YBX1 undergoes phosphorylation by kinases, including AKT and p90 ribosomal S6 kinase, which triggers its rapid translocation into the nucleus [36, 37]. Then YBX1 binds to the promoter regions of specific genes, such as p53, BCL2, and PD-L1, and facilitates their transcription [38, 39]. Interestingly, we noticed that knockdown of EPS8L2 inhibited YBX1 phosphorylation (active state) without affecting its total expression, and vice versa (Fig. 5G). As EPS8L2 is not a kinase, it is imperative to explore the underlying mechanism by which EPS8L2 regulates the phosphorylation status of YBX1. Previous research has demonstrated that the phosphorylation of YBX1 can be mediated by multiple kinases, such as S6K1 [40, 41]. Really, immunoprecipitation assay revealed that knockdown of EPS8L2 significantly decreased the interaction between p-S6K1 and YBX1 (Fig. 5H), suggesting that EPS8L2 facilitated the phosphorylation of YBX1 through S6K1. Moreover, we observed a decrease in nuclear YBX1 following EPS8L2 downregulation and an increase in nuclear YBX1 after EPS8L2 upregulation (Fig. 5I, J).

### YBX1 initiates G3BP2 transcription to activate MAPK signaling pathway

**Fig. 6:**
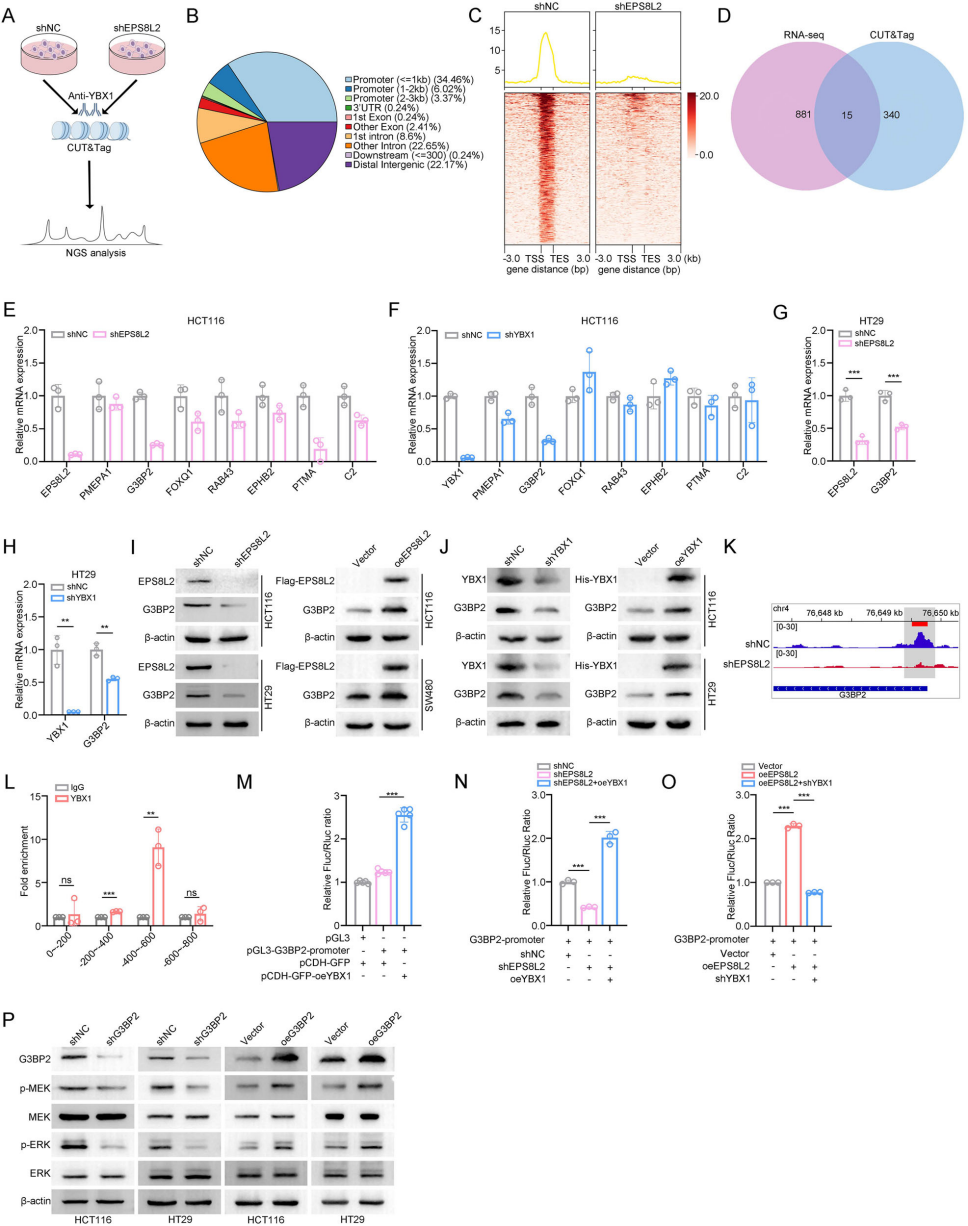
YBX1 initiates G3BP2 transcription to activate MAPK signaling pathway. **A** Schematic representation of CUT&Tag experimental design. **B** Distribution of YBX1 binding sites across the genome, obtained from the comparison between the shNC and shEPS8L2 groups, was shown in pie chart. **C** YBX1 binding intensity around transcription start sites (TSS) and transcription end sites (TES) was displayed by heatmaps and line plots. The red vertical bars typically represent binding strength. **D** The overlap between DEGs identified by RNA-seq and genes with altered YBX1 binding sites identified by CUT&Tag analysis was shown in venn diagram. **E**, **F** Expression of common DEGs was detected by RT-qPCR after EPS8L2 knockdown (**E**) and YBX1 knockdown (**F**) in HCT116 cells. **G**, **H** Expression of G3BP2 was detected by RT-qPCR after EPS8L2 knockdown (**G**) and YBX1 knockdown (**H**) in HT29 cells. **I**, **J** Expression of G3BP2 was detected by western blotting after knockdown or overexpression of EPS8L2 (**I**) and YBX1 (**J**) in CRC cells. **K** YBX1 binding at the G3BP2 promoter region in HCT116 cells was shown by Integrative Genomics Viewer (IGV) images. **L** ChIP-qPCR confirmed the binding of YBX1 to the promoters of G3BP2. **M**–**O** G3BP2 promoter activity was detected by dual-luciferase reporter assay in the indicated HEK293T cells. **P** Expression of G3BP2, p-MEK, MEK, p-ERK, and ERK in G3BP2 knockdown and overexpression CRC cells was detected by western blotting. Data are presented as means ± SD. **p < 0.01, ***p < 0.001, ns no significance.

To further explain how EPS8L2-YBX1 axis activates MAPK signaling pathway, we utilized CUT&Tag technology to identify the direct target of YBX1 (Fig. 6A). The CUT&Tag results indicated that YBX1 binding sites were predominantly distributed in promoter regions, with 34.46% of binding sites located within 1 kb of the transcription start site (TSS) (Fig. 6B). Additionally, YBX1 exhibited higher binding peaks near both the TSS and TES in shNC group, whereas these peaks were significantly diminished in shEPS8L2 group (Fig. 6C). Next, we analyzed the data from the RNA-seq and CUT&Tag experiments jointly and took the intersection to identify the potential targets of YBX1 (Fig. 6D). 15 common genes were identified and their expression levels in CRC were analyzed using the GEPIA online tool (Fig. 6D and Supplementary Fig. 4A–O). Among them, only 7 genes were upregulated in CRC (Supplementary Fig. 4A–G). Therefore, these 7 genes were selected for further investigation by RT-qPCR. The results indicated that the RNA levels of G3BP2 in HCT116 and HT29 cells were significantly reduced following the knockdown of either EPS8L2 or YBX1 (Fig. 6E–H). Consistently, the protein levels of G3BP2 were also regulated by EPS8L2 and YBX1 in a similar manner (Fig. 6I, J).

To determine whether EPS8L2-YBX1 axis directly regulates G3BP2 transcription, we analyzed the enrichment of YBX1 on G3BP2 promoter according to the CUT&Tag result and found that YBX1 enriched across the proximal promoter region (0 bp ~ −850 bp upstream of TSS) of G3BP2 while EPS8L2 knockdown significantly attenuated it (Fig. 6K), indicating an EPS8L2-dependent chromatin recruitment of YBX1. To further confirm it, we performed chromatin immunoprecipitation (ChIP) assay and found that YBX1 enriched on the region of −400 bp ~ −600 bp from TSS (Fig. 6L and Supplementary Fig. 4P). Subsequently, we performed dual-luciferase reporter assays. Consistently, YBX1 overexpression enhanced G3BP2 transcription activity (Fig. 6M). Besides, EPS8L2 knockdown inhibited the activity of G3BP2 luciferase reporter while YBX1 overexpression reversed it, and vice versa (Fig. 6N, O).

As G3BP2 is a member of the RAS-GTPase-activating protein (G3BP) family [42] and G3BP2 is reported to activate the MAPK signaling pathway [43], we hypothesized that G3BP2 may influence the activity of MAPK signaling pathway in CRC. Western blotting showed that the phosphorylation of MEK and ERK was significantly reduced after G3BP2 knockdown and vice versa (Fig. 6P). In conclusion, EPS8L2 promotes YBX1-dependent transcription of G3BP2, leading to MAPK signaling activation.

### EPS8L2 promotes the development of CRC in a G3BP2-dependent manner

**Fig. 7:**
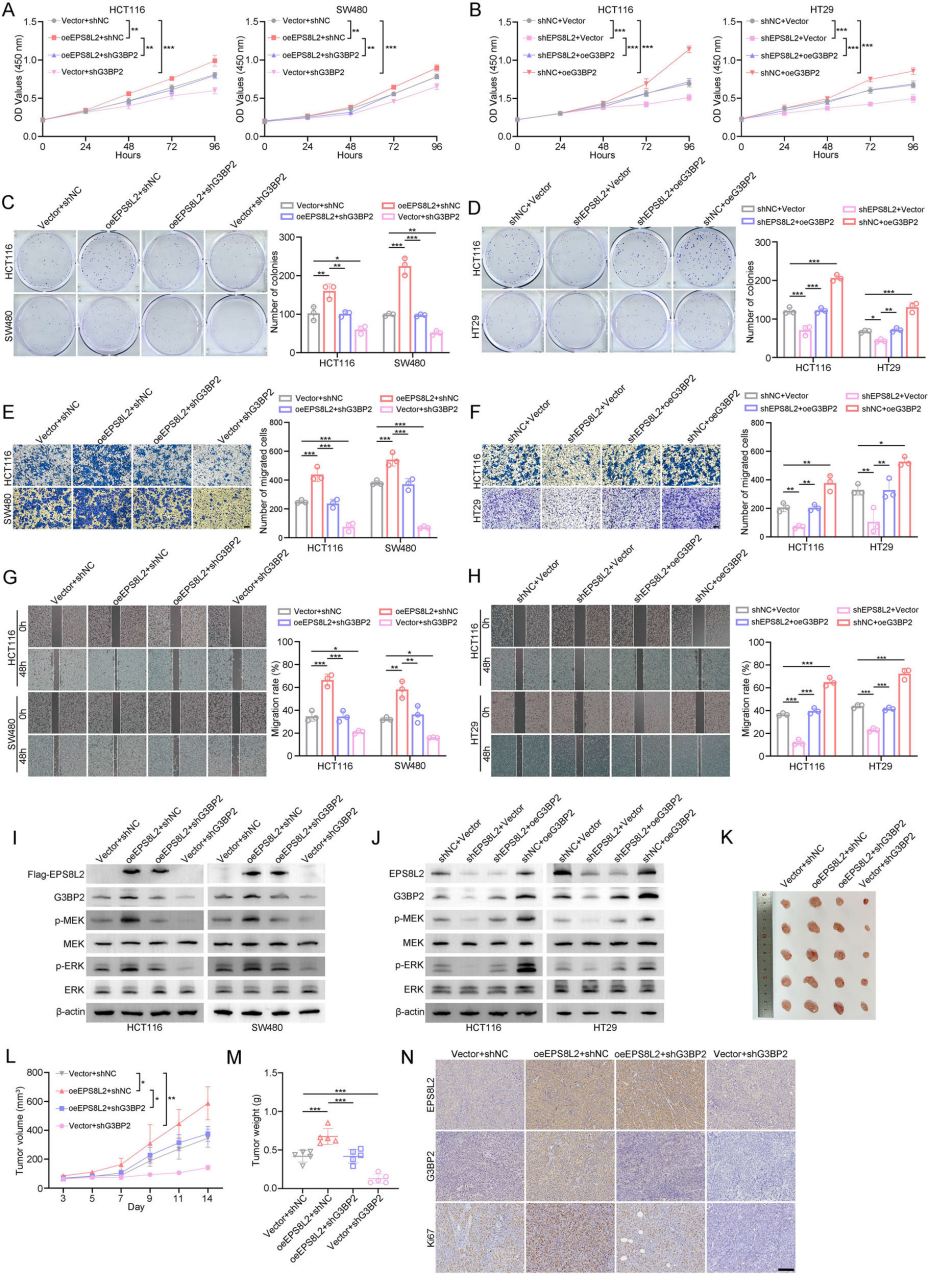
EPS8L2 promotes the progression of CRC in a G3BP2-dependent manner. **A**–**D** Cell proliferation was detected by CCK-8 assay (**A, B**) and colony formation assay (**C, D**) in the indicated CRC cells. **E-H** Transwell migration (**E**, **F**) and wound healing assay (**G, H**) were performed to assess the cell migration ability of indicated CRC cells. Scale bars, 100 μm. **I**, **J** Expression of EPS8L2, G3BP2, p-MEK, MEK, p-ERK, and ERK was detected by western blotting in the indicated CRC cells. **K**–**M** Indicated HCT116 cells were injected into BALB/c nude mice for 14 days (*n* = 5 per group). Images of subcutaneous xenograft tumors (**K**), statistical results of tumor volumes (**L**), and statistical results of tumor weights (**M**) were shown. **N** Expression of EPS8L2, G3BP2, and Ki67 were detected by IHC staining in indicated subcutaneous xenograft tumors. Scale bars, 100 μm. Data are presented as means ± SD. ***p < 0.001, **p < 0.01, *p < 0.05.

To explore whether the ability of EPS8L2 to promote CRC cell proliferation is dependent on G3BP2, we conducted both in vitro and in vivo rescue experiments. The results showed that EPS8L2 overexpression significantly promoted the proliferation and migration abilities of HCT116 and SW480 cells while G3BP2 knockdown reversed it, and vice versa (Fig. 7A–H). Additionally, the activation of MAPK signaling pathway induced by EPS8L2 overexpression could be reversed by G3BP2 knockdown, whereas inhibition of MAPK signaling pathway caused by EPS8L2 knockdown could be restored by G3BP2 overexpression (Fig. 7I, J). In vivo experiments showed that G3BP2 knockdown could impede increased tumor growth and tumor weight caused by EPS8L2 overexpression (Fig. 7K–M). IHC staining results showed that G3BP2 knockdown reversed EPS8L2 overexpression-induced Ki67 upregulation (Fig. 7N). Conclusively, EPS8L2 promotes CRC tumorigenesis and metastasis via G3BP2.

### *Eps8l2* knockout inhibits tumorigenesis in the AOM/DSS model

**Fig. 8:**
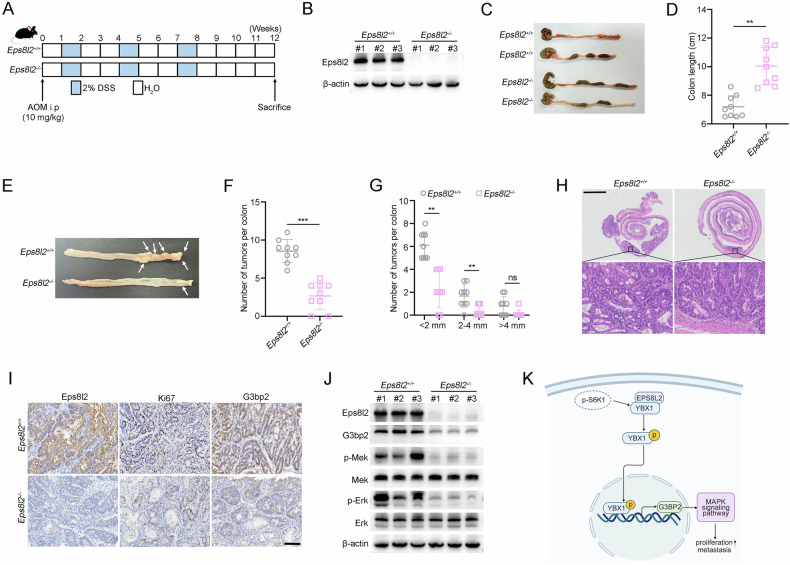
*Eps8l2* knockout inhibits tumorigenesis in the AOM/DSS model. **A** Schematic timeline of AOM/DSS experimental design. **B** Expression of Eps8l2 in colorectal tissues was detected by western blotting. **C** Representative images of colons of *Eps8l2*^+/+^ and *Eps8l2*^−/−^ mice after AOM/DSS treatment (*n* = 9 per group). **D** Colon lengths of *Eps8l2*^+/+^ and *Eps8l2*^−/−^ mice after AOM/DSS treatment were measured. **E** Representative images of colorectal tumors from *Eps8l2*^+/+^ and *Eps8l2*^−/−^ mice after AOM/DSS treatment. Arrows indicate tumors. **F** The number of tumors in *Eps8l2*^+/+^ and *Eps8l2*^−/−^ mice was shown. **G** The number of different size tumors in *Eps8l2*^+/+^ and *Eps8l2*^−/−^ mice. **H** Representative H&E staining of colon tissues obtained from *Eps8l2*^+/+^ and *Eps8l2*^−/−^ mice. Scale bars, 2 mm. **I** Expression of Eps8l2, G3bp2, and Ki67 in tumor tissues was detected by IHC staining. Scale bars, 100 μm. **J** Expression of Eps8l2, G3bp2, p-Mek, Mek, p-Erk, and Erk were detected by western blotting in tumor tissues obtained from *Eps8l2*^+/+^ and *Eps8l2*^−/−^ mice. **K** Schematic model for the findings of this work: EPS8L2 can promote the proliferation and migration of CRC. More importantly, EPS8L2 can enhance the interaction between YBX1 and its kinase p-S6K1, leading to the increase of YBX1 phosphorylation and nuclear translocation, promoting the transcription of downstream G3BP2 and activating the MAPK signaling pathway. Data are presented as means ± SD. ***p < 0.001, **p < 0.01, ns no significance.

To further investigate whether Eps8l2 is involved in tumorigenesis, we generated *Eps8l2* knockout mice (*Eps8l2*^−/−^) and wild-type littermates (*Eps8l2*^+/+^) to induce colorectal cancer (Fig. 8A). We confirmed the deletion of Eps8l2 in the colorectal tissues of *Eps8l2* knockout mice (Fig. 8B). We observed that the length of colons from *Eps8l2*^-/-^ mice was longer than that in *Eps8l2*^+/+^ mice after induction (Fig. 8C, D), suggesting an alleviated inflammation after *Eps8l2* deletion. In addition, the number of colorectal tumors in *Eps8l2*^−/−^ mice was significantly reduced compared with that in *Eps8l2*^+/+^ mice (Fig. 8E, F). Moreover, tumor size was also decreased after *Eps8l2* knockout (Fig. 8G). Consistently, H&E staining revealed low-grade dysplasia in the colon tissues from Eps8l2^-/-^ mice (Fig. 8H). To determine whether the Eps8l2-G3bp2 axis contributes to tumor development in this AOM/DSS model, we examined G3bp2 and Ki67 expression by IHC staining and found a significant decrease of G3bp2 and Ki67 expression levels in tumor tissues from *Eps8l2*^−/−^ mice (Fig. 8I). The levels of p-Mek and p-Erk were significantly reduced in *Eps8l2*^−/−^ tumors (Fig. 8J). Taken together, these data indicate that EPS8L2 is a pivot oncogene involved in CRC progression.

## Discussion

Colorectal cancer persists as a leading cause of global cancer mortality, with American Cancer Society (ACS) data in 2024 reporting approximately 153,000 new cases and 53,000 deaths annually in the US alone [1]. The pathogenesis of CRC is characterized by a complex interplay of multistep genetic and epigenetic alterations, coupled with dysregulation of the tumor microenvironment, which collectively drive malignant progression [44, 45]. In this study, we identified EPS8L2 as a significantly upregulated gene in CRC tumor tissues, where it exhibits oncogenic properties. Clinical correlation analysis revealed that elevated EPS8L2 expression is associated with advanced tumor grade, lymph node metastasis, and unfavorable patient prognosis, suggesting its potential role in CRC progression and aggressiveness. Functional characterization of EPS8L2 revealed its critical role in promoting CRC progression. Furthermore, utilizing patient-derived colorectal cancer organoids as a clinically relevant model system, we demonstrated that EPS8L2 overexpression markedly enhanced organoid formation efficiency, reinforcing its functional role in promoting tumorigenic capacity. Both in vitro and in vivo experiments demonstrated that EPS8L2 overexpression significantly enhanced proliferative and metastatic capacities of CRC cells. Conversely, knockout of Eps8l2 markedly attenuated tumor development in an AOM/DSS-induced CRC mouse model. Collectively, our findings position EPS8L2 as both a potential diagnostic biomarker and a novel therapeutic target for colorectal cancer intervention. Interestingly, we observed that EPS8L2 exerts more significant oncogenic effects in vivo, which could be attributed to the following factor. The tumor microenvironment (TME) in vivo provides complex cell-cell interactions, extracellular matrix components, and immune modulation that are absent in vitro. EPS8L2 may enhance tumor growth by influencing stromal crosstalk and angiogenesis, or reshaping the immune microenvironment in vivo. Secondly, the longer duration and systemic action of EPS8L2 in vivo resulted in a more significant cumulative effect of its tumorigenic activity compared to short-term in vitro experiments. Therefore, it will be worthwhile to explore the relationship of EPS8L2 with the tumor microenvironment and tumor immunity.

MAPK signaling cascade serves as a central regulator of essential cellular processes, governing proliferation, differentiation, cell survival, and programmed cell death. This highly conserved signaling pathway operates through sequential phosphorylation events mediated by a three-tiered kinase module: MAPKKK, MEK, and MAPK [46]. Pathological dysregulation of the MAPK cascade constitutes a fundamental driver of oncogenesis, with particular relevance to colorectal carcinogenesis. In CRC, constitutive activation of the MAPK/ERK signaling axis has been mechanistically linked to multiple malignant phenotypes, including sustained tumor proliferation, metastatic dissemination, and the development of therapeutic resistance [47, 48]. Integrative analysis of transcriptomic sequencing data and correlation studies revealed a significant association between EPS8L2 and MAPK signaling pathway activation in CRC. Functional characterization demonstrated that EPS8L2 serves as a potent activator of MAPK signaling, mechanistically contributing to CRC progression and metastatic dissemination through this oncogenic pathway.

YBX1, a multifunctional transcription factor, regulates critical processes including cell proliferation, differentiation, and apoptosis [38, 49]. Emerging evidence has established YBX1 as a multifunctional oncoprotein contributing to tumorigenesis across multiple cancer types [50–52]. Notably, S6K1-mediated phosphorylation facilitates YBX1 nuclear translocation, thereby activating PD-L1 transcriptional upregulation in HCC [40]. So, we identified YBX1 as a novel interaction partner of EPS8L2 that mediates MAPK pathway activation. Second, we demonstrated that EPS8L2 facilitates the formation of a molecular complex between YBX1 and its activating kinase p-S6K1, thereby promoting YBX1 phosphorylation and subsequent nuclear translocation. However, there is a limitation about above conclusion. We did not perform fluorescence recovery after photobleaching (FRAP) experiments to dynamically monitor YBX1 nuclear accumulation in response to EPS8L2 modulation. Thus, the observed accumulation of YBX1 may also be caused by other factors rather than nuclear translocation, which needs to be defined in the future.

G3BP2, a conserved RNA-binding protein within the RAS-GAP SH3 domain-binding protein (G3BPs) family [42], plays established roles in tumorigenesis and inflammatory diseases [30, 42]. Our CUT&Tag analysis revealed that G3BP2 is co-regulated downstream of EPS8L2 and YBX1. Previous study reported that G3BP2 may function as an oncogene in CRC [53]. Another study indicated that G3BP2 could enhance radioresistance of colorectal cancer cells [54]. Our functional studies demonstrated that G3BP2 knockdown significantly attenuated EPS8L2-driven proliferative and metastatic phenotypes. Moreover, knockdown of G3BP2 effectively suppressed EPS8L2-mediated tumor growth in vivo, suggesting that G3BP2 is a critical downstream effector of EPS8L2 in cancer progression. Notably, these findings align with prior reports that G3BP2 depletion impairs MAPK signaling activation in MCF7 cells, implying a conserved mechanistic role of G3BP2 in oncogenic pathway regulation [43]. Our mechanistic investigations revealed that knockdown of G3BP2 effectively abolished MAPK pathway activation induced by EPS8L2 overexpression, and vice versa. These findings establish G3BP2 as an essential molecular effector in EPS8L2-driven oncogenic transformation.

In conclusion, our study establishes EPS8L2 as a critical oncogenic driver in colorectal cancer progression and metastasis. Mechanistically, we demonstrate that EPS8L2 physically interacts with YBX1 and promotes its nuclear accumulation through phosphorylation-dependent regulation, consequently enhancing G3BP2 transcription and activating the MAPK signaling pathway. Importantly, genetic ablation of Eps8l2 significantly attenuated tumor development in the AOM/DSS-induced CRC model. These findings collectively identify the EPS8L2-YBX1-G3BP2 signaling axis as a novel and potentially targetable pathway for CRC therapeutic intervention.

## Methods

### DEGs, GO, and KEGG analysis

Two GEO datasets (GSE75970 and GSE103512) were downloaded from the Gene Expression Omnibus (GEO: https://www.ncbi.nlm.nih.gov/geo). The TCGA+GTEx dataset was obtained from the TCGA and GTEx databases. DEGs analysis was performed using limma v3.56.2 package in R. The selection criteria for DEGs were |log_2_FC| ≥ 0.58 and *p* < 0.05. The RTK-related genes list was downloaded from Genecards websites. DEGs in three datasets were visualized by volcano plots, and overlap of DEGs across these datasets was shown by venn diagram. 665 common DEGs were subjected to GO and KEGG enrichment analyses using the clusterProfiler package in R. The *p* values were calculated based on the hypergeometric distribution.

### Survival analysis

For survival analysis, according to TCGA database, TPM data and clinical survival data were downloaded from TCGA COAD-READ cohort. Samples were divided into high and low expression groups according to the median value of EPS8L2. Overall survival curves were generated using ggsurvplot function from survminer R package. *P* values were calculated by log-rank test.

For survival analysis, according to CRC tissue array, samples were divided into high and low expression groups according to the IHC score. IHC score above 3 indicated high EPS8L2 expression, while IHC score below 3 indicated low EPS8L2 expression. Survival analysis in different EPS8L2 expression groups was generated using Kaplan–Meier and *p* values were calculated by log-rank test.

### Human specimen

Clinical human colorectal cancer tissues were collected from the First Affiliated Hospital of Zhengzhou University. CRC specimens were collected from the First Affiliated Hospital of Zhengzhou University following informed consent procedures. This study was reviewed and approved by the Institutional Review Board and the Ethics Committee of Zhengzhou University. Additionally, human CRC tissue microarray was purchased from Shanghai Outdo Biotech Company (Shanghai, China).

### Cell culture

Human colorectal cancer cells HCT116, HT29, SW480, and RKO were obtained from the American Type Culture Collection. Human cell line HEK293T (Cat# GNHu17) was purchased from the Cell Bank of the Chinese Academy of Sciences. All cells were cultured in cell incubator (Thermo Fisher Scientific) with 37 °C and 5% CO_2_. All cells were grown in DMEM medium (Viva, #C3113-0500). All media were supplemented with 10% fetal bovine serum (FBS, Lonsera, #S711-001S) and 1% Penicillin-Streptomycin antibiotics solution (Beyotime, #C0222). All cell lines were validated by STR profiling and confirmed to be free from mycoplasma contamination.

### Knockdown and overexpression plasmid construction

To generate stable knockdown CRC cell lines targeting EPS8L2, YBX1, and G3BP2, we employed the pLKO.1-puro lentiviral vector. Gene-specific shRNA sequences were designed using the Sigma-Aldrich online tool (https://www.sigmaaldrich.cn/CN/zh/semiconfigurators/shrna?activeLink=productSearch) and subsequently cloned into the pLKO.1-puro backbone.

For overexpression plasmid construction, coding sequences of human EPS8L2 and G3BP2 were cloned into lentiviral vector pCDH-CMV-MCS-EF1-RFP-T2A-Puro (Tsingke, China) to generate EPS8L2 or G3BP2 overexpression plasmids. Coding sequences of human YBX1 were cloned into lentiviral vector pCDH-copGFP-puro (Tsingke, China) to generate YBX1 overexpression plasmids. When constructing the EPS8L2 and YBX1 overexpression vector, we added a Flag and His tag to the 3’ end of the CDS, respectively. Overexpression primers and shRNA primers were synthesized by Sangon Biotech (Shanghai, China). The primer sequences of EPS8L2, YBX1, and G3BP2 shRNA and CDS were listed in Supplementary Tables 1 and 2, respectively.

### Lentiviral infection

The knockdown or overexpression plasmid and control plasmid were transfected into HEK293T cells with lentiviral packaging plasmids psPAX2 (Addgene, #12260) and pMD2.G (Addgene, #12259) according to protocol of jetPRIME reagent (Polyplus, #101000046). Virus medium was filtered through a 0.45-micron filter and subsequently mixed 1:1 with complete medium for culturing target cells. Polybrene (Yeasen, #40804ES86) was added to enhance infection. After 24 h, the medium was replaced with fresh complete medium. The infected cells were subjected to puromycin selection (0.8 μg/mL for HCT116 cells, 2 μg/mL for HT29 cells, 1 μg/mL for SW480 cells, and 1 μg/mL for RKO cells) for 3–4 days to establish stable knockdown or overexpression cell lines. RT-qPCR and western blotting were performed to detect EPS8L2 knockdown or overexpression efficiency.

### RNA isolation and quantitative real-time PCR (RT-qPCR)

Total RNA from whole cell lysates or tissues was isolated by Total RNA Extraction Reagent (Vazyme, #R711-01). The concentration and purity of total RNA were determined by NanoDrop2000 (Thermo Fisher, USA). The HiScript III RT SuperMix for RT-qPCR (+gDNA wiper) (Vazyme, #R323) was used for reverse transcription reaction. RT-qPCR was peformed on CFX96TM Real-Time System (Bio-RAD, #CT032238) using Hieff^®^ qPCR SYBR Green Master Mix (No Rox) (Yeasen, 11201ES03). Data analysis was used 2^−ΔΔCt^ method and normalized to β-actin. The primer sequences were presented in Supplementary Table 3.

### Western blotting

Samples were lysed using RIPA lysis buffer (Beyotime, #P0013B) containing protease inhibitor PMSF (Solarbio, #P0100), and extracted protein was quantified using BCA assay (Beyotime, #P0012). The proteins were separated using SDS-polyacrylamide gel electrophoresis (PAGE) and transferred to polyvinylidene fluoride (PVDF) membrane (Merck Millipore, #IPVH00010). The membranes were blocked with 5% non-fat milk for 2 h at room temperature, followed by incubation with primary antibody overnight at 4 °C. The next day, the membranes were incubated with secondary antibody for 1 h at room temperature. Protein expression was visualized using BeyoECL Star (Beyotime, #P0018AS) by enhanced chemiluminescent system BG-gds AUTO 720 (Baygene Biotech, China). β-actin was used as the internal reference protein. The antibodies were listed in Supplementary Table 4.

### Cell counting kit-8 (CCK-8) assay

Cell viability was assessed by CCK-8 assay (APExBIO, #K1018). Cells of logarithmic growth phase were seeded into 96-well plates at a density of 1 × 10^3^ cells in 100 μL medium per well. Ten microliters CCK-8 reagent was added to each well at 0, 24, 48, 72, 96 h and incubated at 37 °C for 1 h. The absorbance at 450 nm was measured using microplate reader (Bio-Rad, USA) at corresponding time.

### Colony formation assay

CRC cells (500 cells/well for HCT116 cells, 800 cells/well for HT29 cells, 800 cells/well for SW480 cells, and 700 cells/well for RKO cells) were seeded into 6-well plates and cultured at 37 °C for 8–18 days. Then cells were fixed with 4% paraformaldehyde for 30 min and stained with 0.1% crystal violet for 15 min. Colony numbers were counted and recorded by Image J software.

### Transwell migration assay

The migration assay was performed using transwell chambers (BIOFIL, TCS003012). CRC cells (5 × 10^4^ cells/well for HCT116 cells, 2 × 10^5^ cells/well for HT29 cells, 1 × 10^5^ cells/well for SW480 cells, and 2 × 10^5^ cells/well for RKO cells) were seeded into the upper chamber with serum-free DMEM, and then 20% FBS DMEM was added to the lower chamber. After 48 h, CRC cells were fixed in 4% paraformaldehyde for 30 min and stained with 0.1% crystal violet for 15 min. Images were captured using the Eclipse Ti2 microscope (Nikon, Japan). Migratory cells were quantified using ImageJ software.

### Wound healing assay

CRC cells were seeded in 6-well plates and allowed to grow until reaching confluency. Ten microliters pipette tip (Axygen, #AXYT300) was used to create a scratch in the confluent cell layer. After 48 h, images of cells migrating into scratched area were captured using inverted microscope (Eclipse Ti2, Nikon, Japan). The migration distance was measured, and the migration rate was calculated with the following formula: (Width at 0 h − Width at 48 h)/Width at 0 h.

### Apoptosis assay

The apoptotic rate of CRC cells was detected using apoptosis detection kit (Yeasen, #40302ES60). Cells were treated with EDTA-free trypsin and then collected by centrifugation at 300 × g for 5 min at 4 °C. After cells were washed twice with pre-cooled PBS, 100 μL 1×Binding Buffer was added to resuspend cells. Then, 10 μL PI staining solution and 5 μL Annexin V-FITC were added, and the mixture was gently mixed. The reaction was carried out in the dark at room temperature for 10–15 min. After 400 μL 1×Binding Buffer was added to each tube, mixture was gently mixed and prepared to flow cytometry analysis.

### Animal experiments

All animal experiments were performed at the Laboratory Animal Center of the Zhengzhou University and approved by the Ethics Committee of Zhengzhou University (ZZUIRB2023-320, 15 December 2023). Four-week-old female BALB/c nude mice were purchased from SPF Biotechnology Co., Ltd. (Beijing, China) and then housed in barrier system. No statistical methods were applied to choose the number of mice. For this analysis; blinding was not conducted. Mice were randomly divided into appropriate groups based on the experimental design. For EPS8L2 knockdown subcutaneous tumor model, HCT116 cells (6 × 10^6^ per mouse) in 100 μL were subcutaneously transplanted into nude mice in both the shNC group and the shEPS8L2 group (*n* = 6 per group). For EPS8L2 overexpression subcutaneous tumor model, HCT116 cells (5 × 10^6^ per mouse) in 100 μL were subcutaneously injected into nude mice in both the vector group and the oeEPS8L2 group (*n* = 4 per group). For rescue subcutaneous tumor model, HCT116 cells (5 × 10^6^ per mouse) in 100 μL were subcutaneously injected into nude mice (*n* = 5 per group). After the subcutaneous tumors became detectable, tumor size was measured every other day, and the tumor volume was calculated using the formula: volume (mm^3^) = length × width^2^/2. The tumor tissues were used for western blotting, RT-qPCR, and IHC.

For EPS8L2 knockdown tail vein metastasis model, the mice were randomly divided into shNC and shEPS8L2 groups. Each mouse was injected with 5 × 10^6^ cells in 100 μL sterile PBS via the tail vein and observed for 33 days (*n* = 4 per group). For EPS8L2 overexpression tail vein metastasis model, the mice were randomly divided into vector and oeEPS8L2 groups. Each mouse was injected with 3 × 10^6^ cells in 100 μL sterile PBS via the tail vein and observed for 28 days (*n* = 4 per group). The mice were sacrificed at the experimental endpoint, and the livers were collected and photographed. The number of liver metastatic nodules was counted. The liver tissues were fixed in 4% paraformaldehyde for 24 h and embedded in paraffin for hematoxylin and eosin (HE) staining.

For AOM/DSS model, *Eps8l2* KO mice were obtained from Cyagen biosciences (KOAI231030WX1, China) and confirmed by genotyping. 7-week-old female mice (*n* = 9 per group) were treated with AOM (Sigma, #A5486-25mg) at 10 mg/kg in PBS via intraperitoneal injection on Day 0. Seven days later, 2% DSS was given in drinking water for 7 days, followed by regular water for two weeks. After three cycles of DSS treatment, the mice were sacrificed 84 days after AOM injection. Mice colon length was measured, and visible tumors were counted. Finally, the colons were photographed and collected for subsequent analysis. The genotyping primers are listed in Supplementary Table 5.

### Immunohistochemistry (IHC) staining and scoring

Human or mouse tissue samples were fixed in 4% paraformaldehyde and embedded in paraffin. Before staining, the sections were deparaffinized in xylene and rehydrated in 100%, 95%, and 80% alcohols. Tissue sections were incubated with 3% hydrogen peroxide solution for 15 min to inhibit endogenous peroxidase activity. Subsequently, they were blocked with 10% Donkey Serum (Solarbio, #SL050) at room temperature for 45 min. Antigen retrieval was performed by heating the slices in antigen retrieval solution at 100 °C for 15 min. Next, the sections were incubated overnight at 4 °C with primary antibody. The next day, they were incubated with secondary antibody for 1 h at room temperature. Then, 3,3′-Diaminutesobenzidine (DAB) Horseradish Peroxidase Color Development Kit (ZSGB-BIO, #ZLI9019) was used as chromogen, and hematoxylin was applied for nuclear counterstain. Finally, the sections were mounted using neutral balsam after dehydration. The slides were imaged using GCell ImageViewer (GCell, China). Well-stained areas were selected to observe the staining intensity and stained area of positive cells under the microscope. The score of intensity was categorized into four levels: negative (0), weak (1), moderate (2), and strong (3). The positive cell percentage was divided into five levels 0% (0), 0–25% (1), 26–50% (2), 51–75% (3), and 76–100% (4). The final staining score was the sum of the staining intensity and staining area scores, with a range of 0 to 7. The antibodies were listed in Supplementary Table 4.

### RNA-sequencing (RNA-seq) and analysis

EPS8L2 knockdown (shEPS8L2-1) and control HCT116 (shNC) cells were used for transcriptome sequencing. Total RNA was extracted using Total RNA Extraction Reagent (Vazyme, #R711-01), and the purity and concentration of RNA were measured using NanoDrop2000 spectrophotometer (Thermo Fisher, USA). The RNA samples were sent to Berry Genomics for RNA sequencing. The Illumina NovaSeq6000 sequencing platform (Illumina, USA) was used for sequencing. DEGs were defined using the following threshold: |log_2_FC| ≥ 0.56 and *p* < 0.05. The clusterProfiler R package was used for GO and KEGG enrichment analysis of DEGs.

### Immunoprecipitation and mass spectrometry

The cells were collected and lysed using RIPA lysis buffer (Beyotime, #P0013B) containing protease inhibitor PMSF (Solarbio, #P0100). Meanwhile, Protein A/G agarose beads (Engibody, #IF0001) were prewashed with RIPA lysis buffer. For each sample, 30 μL pre-washed beads were added and incubated at 4 °C for 1 h to remove non-specific proteins. The samples were centrifuged at 13,000 rpm for 15 min at 4 °C, then the supernatant was collected and transferred to a new tube. The cell lysates were then incubated with specific antibodies overnight at 4 °C, then the pre-washed beads were added into the mixture for 4 h at 4 °C. After 2500 rpm for 5 min at 4 °C centrifugation, the supernatant was discarded. 2× SDS loading was added into the beads and boiled for 15 min to get samples. For co-IP/MS, IgG or Flag antibody was added into HCT116 and SW480 cell lysates. Immunocomplexes were subjected to SDS-PAGE separation. Then, the gels were stained with the silver staining kit (Beyotime, #P0017S), and differential bands were cut off for MS analysis. MS analysis was performed by Oebiotech (Shanghai, China).

### Nuclear-cytoplasmic separation assay

The nuclear-cytoplasmic separation assay was performed according to Nuclear/Cytosol Fractionation Kit (Phygene, #PH1466) manufacturer’s protocol. Briefly, the CRC cell lines were collected into centrifuge tubes. The pellets were washed twice with pre-cooled PBS, centrifuged at 500 × *g* at 4 °C for 3 min after each wash. Next, 400 μL pre-cooled extraction buffer A was added to the cell pellet, and the mixture was incubated on ice for 30 min with vertexing every 5 min to ensure thorough mixing. After shaking and mixing, the samples were centrifuged at 12,000 × *g* at 4 °C for 5 min. The supernatants that contained cytoplasmic proteins were transferred to precooled centrifuge tubes, while the pellets were resuspended in pre-cooled PBS and washed 3 times. The remaining pellet was nuclear fraction. RIPA lysis buffer with protease inhibitor PMSF was added to cytoplasmic fraction and nuclear fraction for 30 min on ice. The concentrations of cytoplasmic and nuclear proteins were measured using the BCA assay and analyzed by western blotting.

### CUT&Tag sequencing and analysis

EPS8L2 knockdown (shEPS8L2-1) and control HCT116 (shNC) cells were used for CUT&Tag assay according to the protocol of Hyperactive Universal CUT&Tag Assay Kit for Illumina Pro (Vazyme, #TD904-01/02). 2 × 10^5^ cells were harvested for each reaction. Anti-YBX1 antibody (Huabio, # ET1609-10, 1:50) was used as primary antibodies. Libraries were tested by Agilent 2100 bioanalyzer. All CUT&Tag libraries were sequenced by Novogene using Illumina NovaSeq XPlus platform in PE150 mode. Quality control of the raw CUT&Tag data was performed using FastQC, followed by low-quality base trimming from the fastq files using Trim_Galore v0.6.10. The data were aligned to the reference genome using Bowtie2. Paired-end reads were converted into paired BED files using samtools view, and duplicate reads were removed using samtools rmdup for downstream analysis. Peak calling was conducted using MACS2 v2.2.7.1. Differential peak analysis between groups was performed using Macs2 bdgdiff, and peaks were visualized and rendered using Integrative Genomics Viewer (IGV v2.16.0). Each peak was annotated using bedtools intersect based on GENCODE gene annotation. Heatmap were generated using deepTools computeMatrix.

### Luciferase reporter assay

The promoter sequence of G3BP2 was cloned into pGL3-basic vector. Luciferase reporter plasmid, shEPS8L2, oeEPS8L2, shYBX1, oeYBX1 plasmid, or negative control plasmid were co-transfected into HEK293T cells using jetPRIME transfection reagent. After 48 h, the cells were lysed and prepared according to Dual Luciferase Reporter Gene Assay Kit (Beyotime, #RG027). Renilla luciferase activity as an internal control. The primers are listed in Supplementary Table 2.

### Chromatin Immunoprecipitation assay

2 × 10^7^ HEK293T cells were collected and cross-linked with 1% formaldehyde at room temperature for 10 min. Next, quenched with 2.5 M glycine at room temperature for 5 min and lysed in SDS lysis buffer on ice for 10 min. Chromatin was sonicated (200 W, 30 s on/60 s off, 5 cycles) and fragment size verified by agarose gel electrophoresis. After centrifugation at 14,000 rpm at 4 °C for 10 min, the supernatant was diluted 10-fold in ChIP dilution buffer. Non-specific binding was pre-cleared with Protein A/G beads 50 μL at 4 °C for 1 h. The pre-cleared lysate was divided equally: one half incubated with control IgG (2 μg), the other with anti-YBX1 antibody (2 μg) at 4 °C for overnight. 50 μL Protein A/G beads were added at 4 °C for 4 h to capture immunocomplexes. Beads were washed sequentially with low salt, high salt, LiCl, and TE buffers. Bound complexes were eluted with Elution Buffer 500 μL at room temperature for 15 min. Eluates were de-crosslinked with 5 M NaCl at 65 °C for 4 h, and proteins digested with proteinase K at 45 °C for 2 h. DNA was purified by phenol-chloroform and dissolved in nuclease-free water for qPCR analysis using primers from Supplementary Table 6.

### Patient-derived organoids (PDOs) isolation and treatment

Freshly resected tumor tissue was immediately placed in ice-cold RPMI-1640 medium and processed within 30 min. Superficial mucosa was carefully removed using scissors. The tissue was then washed vigorously 5–10 times with HBSS until blood residues were removed. The washed tissue was minced finely with scissors in 5 mL tube, transferred to 15 mL centrifuge tube, and digested with 6 mL of pre-warmed digestion solution at 37 °C under gentle agitation. Digestion progress was briefly monitored microscopically after 10 min. Digestion was terminated by adding 3 times volume of ice-cold DPBS, followed by vigorous pipetting 20–30 times. The resulting cell suspension was filtered through a 70 μm cell strainer. The filtrate was centrifuged at 300 × *g* for 5 min at 4 °C. The supernatant was discarded, and the cell pellet was resuspended in organoid culture medium (DIMED, K211M01) to an appropriate density. The cell suspension was then mixed with Matrigel™ at a 1:2 ratio. Aliquots of the cell-Matrigel mixture were plated in 48-well plate and solidified for 15 min at 37 °C. Finally, 300 μL of pre-warmed organoid culture medium was added to each well, and cultures were maintained in incubator.

### Immunofluorescence (IF) assay

Cells on coverslips were washed twice with PBS and fixed in 4% paraformaldehyde (PFA) for 10 min at room temperature. After PBS washing, cells were permeabilized with 1% Triton X-100 for 20 min at room temperature and blocked with 10% goat serum for 30 min at room temperature. Subsequently, samples were incubated overnight at 4 °C with primary antibody EPS8L2 and YBX1. Next day, samples were incubated with HRP-conjugated secondary antibody for 1 h at room temperature, followed by Tyramide Signal Amplification (TSA) reaction according to manufacturer’s protocols (Aifang Bio, Cat# AFIHC036). Nuclei were counterstained with DAPI for 5 min at room temperature. Finally, the slides were sealed with anti-fluorescence quenching sealer (Cat# S2100, Solarbio).

### Statistical analysis

The results were analyzed using GraphPad Prism 8 and R 4.3.2. The data from both groups were normally distributed, and *p* values between the two groups were calculated using two-tailed paired or unpaired Student’s t-tests when the variances were similar. Data that did not conform to normal distribution were analyzed using nonparametric tests. The variance dissimilar between groups were analyzed by Welch’s correction. One-way ANOVA was used for multiple group comparisons. Two-way ANOVA was used to calculate the statistical significances of cell proliferation and tumor growth in different groups. *P* values of survival analysis were calculated using log rank tests. Gene correlation analysis in R was assessed using Pearson’s test. The sample size was not determined based on a priori statistical calculations, and no data were excluded during analysis. Each experiment was independently performed at least three times. All values were expressed as mean values ± SD. In all statistical analyses, *p* values of <0.05 were considered statistically significant (*p < 0.05, **p < 0.01, ***p < 0.001, ns indicates no significant difference).

### Accession numbers

Raw RNA-sequence data have been deposited in the NCBI Sequence Read Archive (SUB15382866, accession code: PRJNA1275751).

## Supplementary information

Supplementary materials

uncropped western blots

RT-qPCR original data

## Data Availability

All data are available upon reasonable request.
