# Supplementary material for: EPS8L2 drives colorectal cancer cell proliferation and migration via YBX1-dependent activation of G3BP2 transcription
Source: Cell Death Dis. 2025 Aug 10;16(1):605. doi: 10.1038/s41419-025-07929-x (PMC12335500; doi:10.1038/s41419-025-07929-x)
Supplement: Supplementary file 1 — Supplementary materials [file 41419_2025_7929_MOESM1_ESM.docx]

**
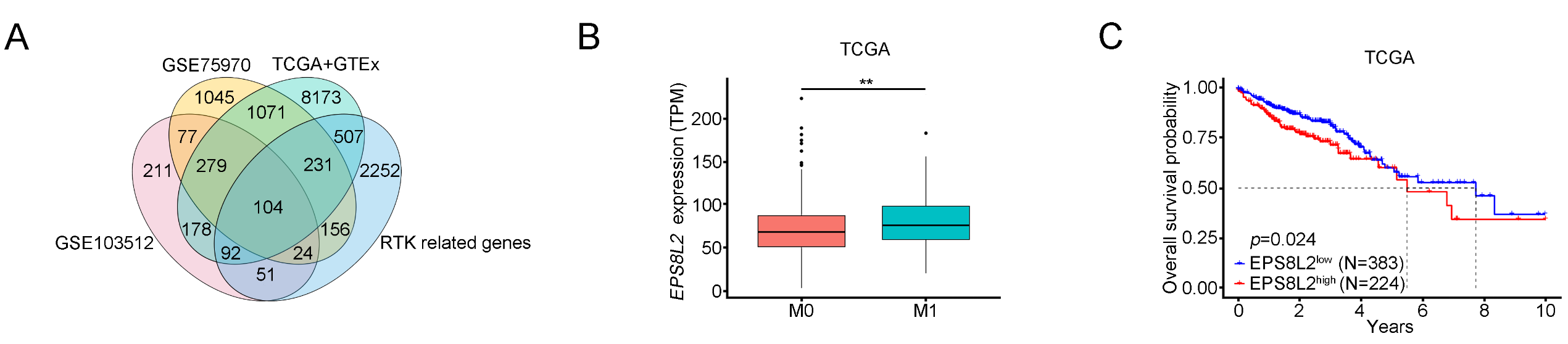
**

**Supplementary Figure 1. EPS8L2 expression and survival analysis in CRC.** (A) RTK related DEGs in figure 1B were displayed by Venn diagram. (B) EPS8L2 expression in CRC non-metastasis tissues and CRC metastasis tissues according to TCGA database. TPM represents transcripts per million. M0 represents patients without distant metastasis and M1 represents patients with distant metastasis. (C) Relationship between EPS8L2 expression and CRC patients’ overall survival according to TCGA database. ***p* < 0.01.


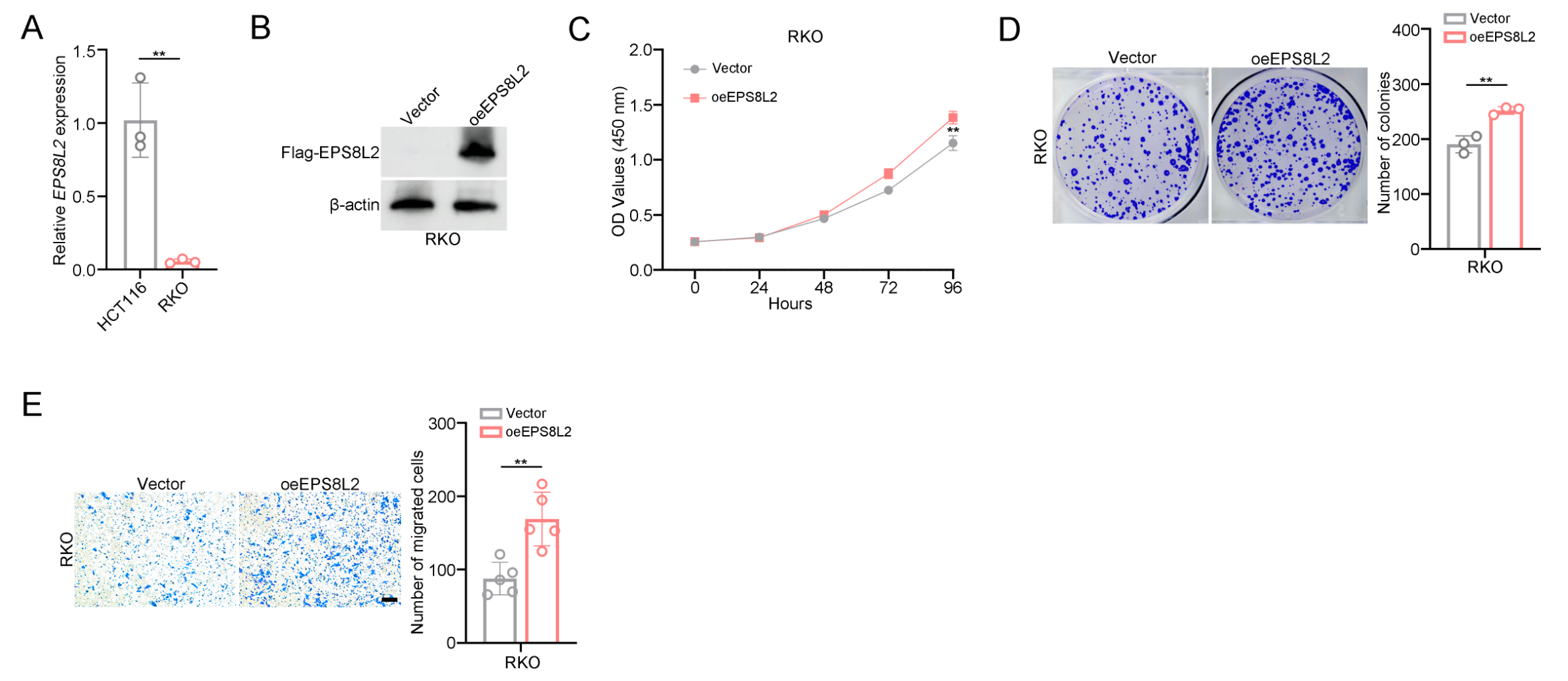


**Supplementary Figure 2. EPS8L2 overexpression promotes proliferation and migration of RKO cells.** (A) EPS8L2 expression were examined by RT-qPCR in HCT116 and RKO cells. (B) EPS8L2 expression were examined by western blotting in EPS8L2 overexpression RKO cells. (C, D) Cell proliferation was measured using CCK-8 assay (C) and colony formation assay (D) after EPS8L2 overexpression in RKO cells. (E) Transwell assays were conducted to assess the migratory ability of RKO cells after EPS8L2 overexpression. Scale bars, 100 μm. Representative images were shown in left panel and statistical results were shown in right panel. Data are presented as means ± SD. ***p* < 0.01.

**
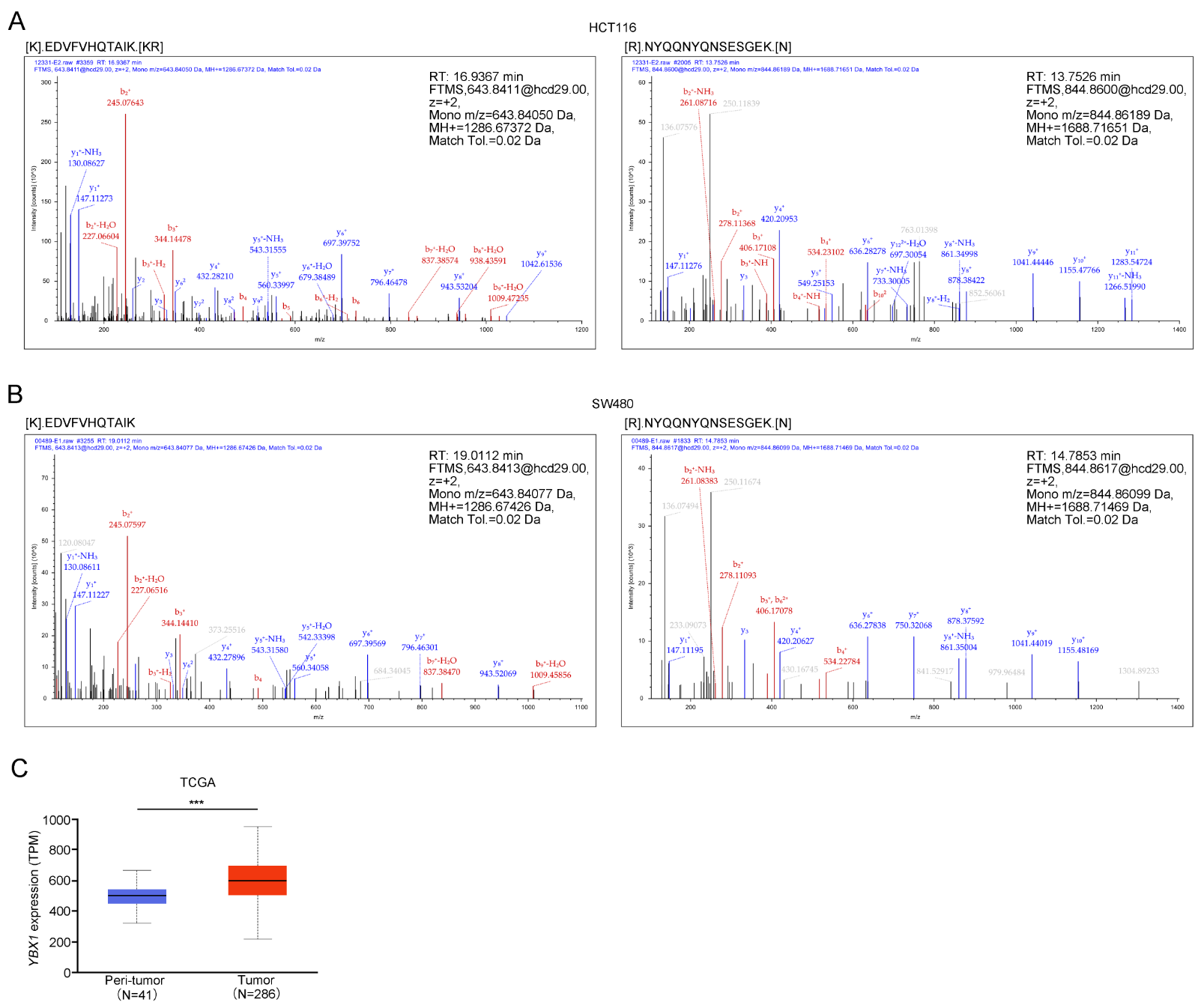
**

**Supplementary Figure 3. EPS8L2 interacts with YBX1.** (A, B) Peptides of YBX1 were identified by mass spectrometry in HCT116 (A) and SW480 (B) cells. (C) YBX1 expression according to TCGA database of ULCAN website. ****p* < 0.001.


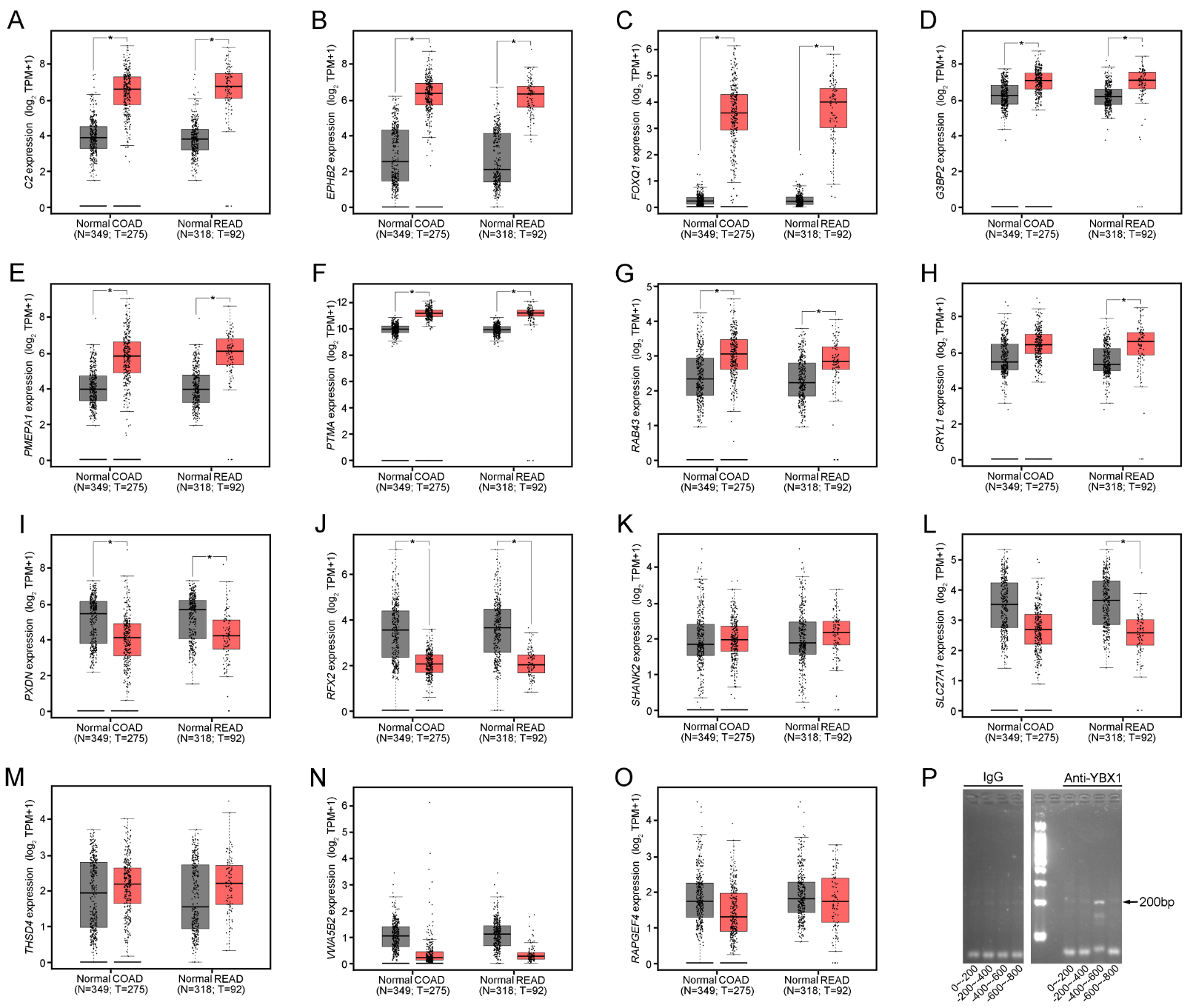


**Supplementary Figure 4. Expression of common DEGs according to GEPIA website.** (A-G) Upregulated expression of common DEGs in COAD and READ according to GEPIA website. (H-O) No differential expression or downregulated expression of common DEGs in COAD and READ according to GEPIA website. COAD: colon adenocarcinoma, READ: rectal adenocarcinoma. Boxplot lower boundary represents the first quartile, the upper boundary represents the third quartile, and the median is indicated by solid horizontal line. (P) Gel electrophoresis results of ChIP-qPCR products. **p* < 0.05.

**Supplementary Table 1. shRNA sequences used in this study.**

| Genes | (5′-3′) |
| --- | --- |
| shEPS8L2-1 | GAACAAGTTTCATTCCATGAA |
| shEPS8L2-2 | GTCCATAAGAAACTCCCAGAA |
| shYBX1 | CCAGTTCAAGGCAGTAAATAT |
| shG3BP2 | GACTCTGACAACCGTAGAATA |

**Supplementary Table 2. Primer sequences for gene and promoter construction.**

| Genes | (5′-3′) |
| --- | --- |
| oeEPS8L2-F | CCGGAATTCATGAGCCAGTCCGGGGCCGTGAG |
| oeEPS8L2-R | CGCGGATCCCTACTTGTCATCGTCGTCCTTGTAATCGCTGTCCTCCCCCCTCCTCTGAT |
| oeYBX1-F | GCTCTAGAATGAGCAGCGAGGCCGAGACCCAG |
| oeYBX1-R | GCGGATCCTTAATGGTGATGGTGATGATGCTCAGCCCCGCCCTGCTCAGCCTCGG |
| oeG3BP2-F | GCTCTAGAATGGTTATGGAGAAGCCCAGTCC |
| oeG3BP2-R | GCGGATCCTCACTTGTCATCGTCGTCCTTGTAATCGCGACGCTGTCCTGTGAAGCGG |
| G3BP2-promoter-F | ATTTCTCTATCGATAGGTACCAATACTATATAAAGTTCCCAAAAAGTATTG |
| G3BP2-promoter-R | ACTTAGATCGCAGATCTCGAGCACGTGGGTGGCTGCGGC |

**Supplementary Table 3. Primer sequences for RT-qPCR.**

| Genes | (5′-3′) |
| --- | --- |
| EPS8L2-F | TCATGGACAAGAGCGAAGCC |
| EPS8L2-R | GCTCCTCCTGTGACTCGATG |
| β-actin-F | CATGTACGTTGCTATCCAGGC |
| β-actin-R | CTCCTTAATGTCACGCACGAT |
| YBX1-F | AAGTGATGGAGGGTGCTGAC |
| YBX1-R | CTAGGCTGTCTTTGGCGAGG |
| G3BP2-F | CCGTTGGCTGGAGCATTTG |
| G3BP2-R | AACAGCTTCCTGGGGCTTTC |
| PMEPA1-F | ACTGCAAACGCTCTTTGTTCC |
| PMEPA1-R | TGATGAAGGACCGTGCAGAC |
| PTMA-F | CACCCAACCCAAACCATGAG |
| PTMA-R | CGAAGGCTGGTTTGGTCATC |
| FOXQ1-F | TCAACGAGTACCTCATGGGC |
| FOXQ1-R | TACTCGCTGTTGGGGTTGAG |
| EPHB2-F | GTGTAACAGAAGACGGGGGT |
| EPHB2-R | GACTTGAGCGTCTTGATGGC |
| RAB43-F | CTTCGGCTTCTTCTAGGCCA |
| RAB43-R | CCAGATCTGCAGCTTGACCC |
| C2-F | TCTGCAAACCTGTGCGCT |
| C2-R | CAAGATTCGAGGAGCAGCGA |

**Supplementary Table 4. The information of antibodies.**

| Antibody | Company | Application |
| --- | --- | --- |
| EPS8L2 | Proteintech (20461-1-AP) | WB 1:2000, IHC 1:500, IF: 1:500 |
| Flag | Sigma-Aldrich (F1804) | WB 1:5000 |
| His | Sigma (SAB1305538) | WB 1:5000 |
| MMP9 | Proteintech (10375-2-AP) | WB 1:1000 |
| MMP2 | Proteintech (10373-2-AP) | WB 1:1000 |
| BAX | Proteintech (50599-2-Ig) | WB 1:2000 |
| BCL2 | Proteintech (12789-1-AP) | WB 1:1000 |
| MEK1/2 | Proteintech (11049-1-AP) | WB 1:10000 |
| p-MEK1/2 | CST (9154S) | WB 1:1000 |
| ERK1/2 | CST (4695S) | WB 1:1000 |
| p- ERK1/2 | CST (4370S) | WB 1:2000, IHC 1:200 |
| YBX1 | Huabio (ET1609-10) | WB 1:2000, IF: 1:500 |
| p-YBX1 | Nature Biosciences (A89296) | WB 1:1000 |
| G3BP2 | Huabio (ER63482) | WB 1:1000 |
| β-actin | Sigma (sc-47724) | WB 1:5000 |
| p-S6K1 | Nature Biosciences (A64169) | WB 1:1000 |
| Ki67 | Abcam (ab16667) | IHC 1:1000 |
| LaminB1 | Nature Biosciences (A60474) | WB 1:1000 |
| Goat Anti-Rabbit IgG Antibody (H+L), HRP Conjugated | ZSGB-bio (ZB-2301) | WB 1:4000, IHC 1:500 |
| Goat Anti-mouse IgG Antibody (H+L), HRP Conjugated | ZSGB-bio (ZB-2305) | WB 1:4000, IHC 1:500 |

**Supplementary Table 5. Primer sequences for mouse genotyping.**

| Genes | (5′-3′) |
| --- | --- |
| Eps8l2-F1 | CACATCAGGCAGTTCATATCTC |
| Eps8l2-R1 | GTCCCTTTCTAACATTGATTAT |
| Eps8l2-F2 | CACATCAGGCAGTTCATATCTC |
| Eps8l2-R2 | GACAGATACATCTCTGTGATGCTGA |

**Supplementary Table 6. Primer sequences for ChIP-qPCR.**

| Genes | (5′-3′) |
| --- | --- |
| 0~-200-F | CAGCAGCGGCCAAGA |
| 0~-200-R | CCACGTGGGTGGCTG |
| -200~-400-F | GCGGAGGAAATTCATCTT |
| -200~-400-R | TCTTGGCCGCTGCTG |
| -400~-600-F | GGATTTCAGGAGCGGAA |
| -400~-600-R | GTAATGGGGGGTCAGAG |
| -600~-800-F | GAAAGAGGGAGGGGC |
| -600~-800-R | CCCAGAGTTATTCAATCACG |
