# Supplementary material for: EPS8L2 drives colorectal cancer cell proliferation and migration via YBX1-dependent activation of G3BP2 transcription
Source: Cell Death Dis. 2025 Aug 10;16(1):605. doi: 10.1038/s41419-025-07929-x (PMC12335500; doi:10.1038/s41419-025-07929-x)

Figure-2B

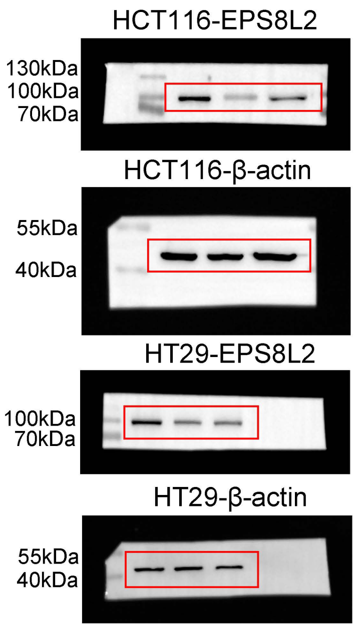

Figure-2H

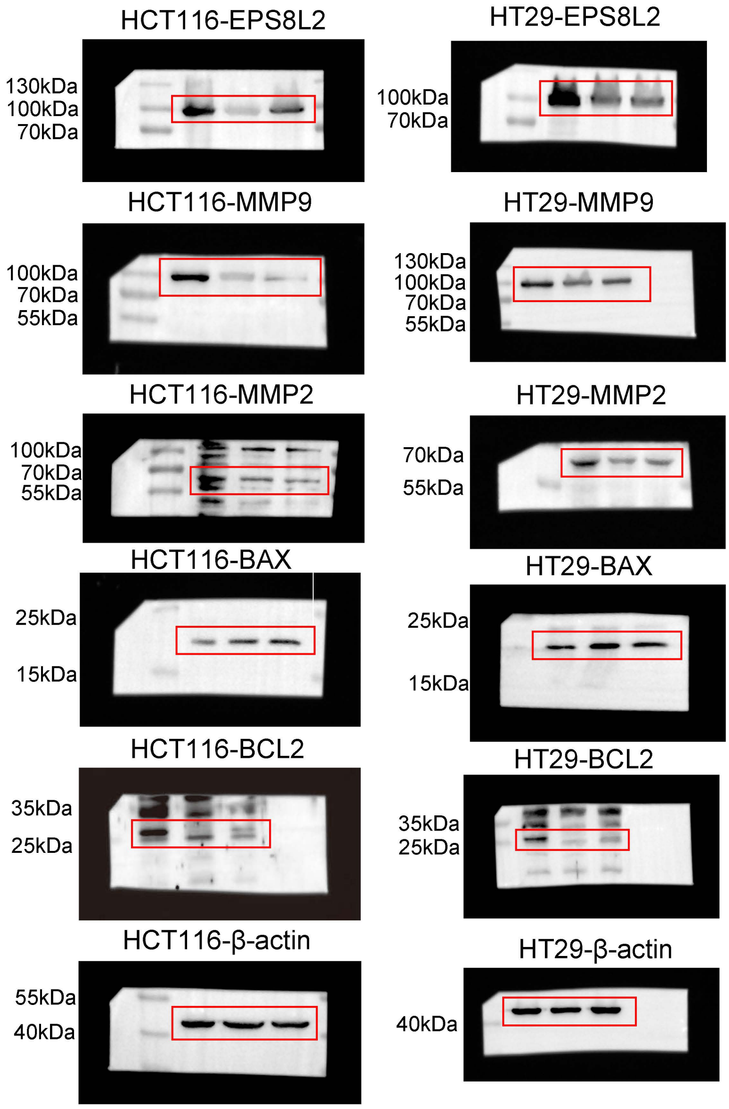

Figure-3B

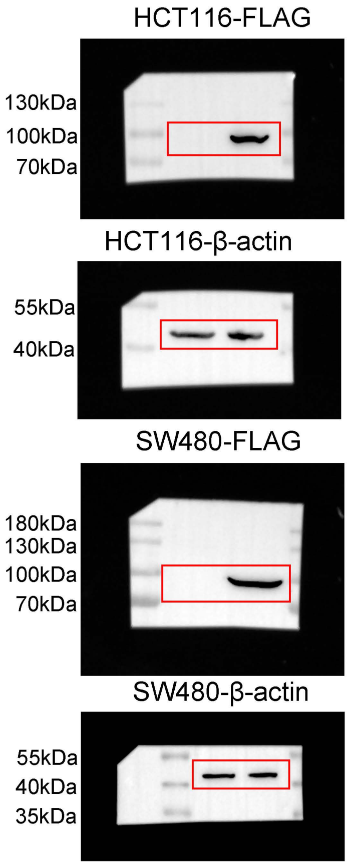

Figure-3G

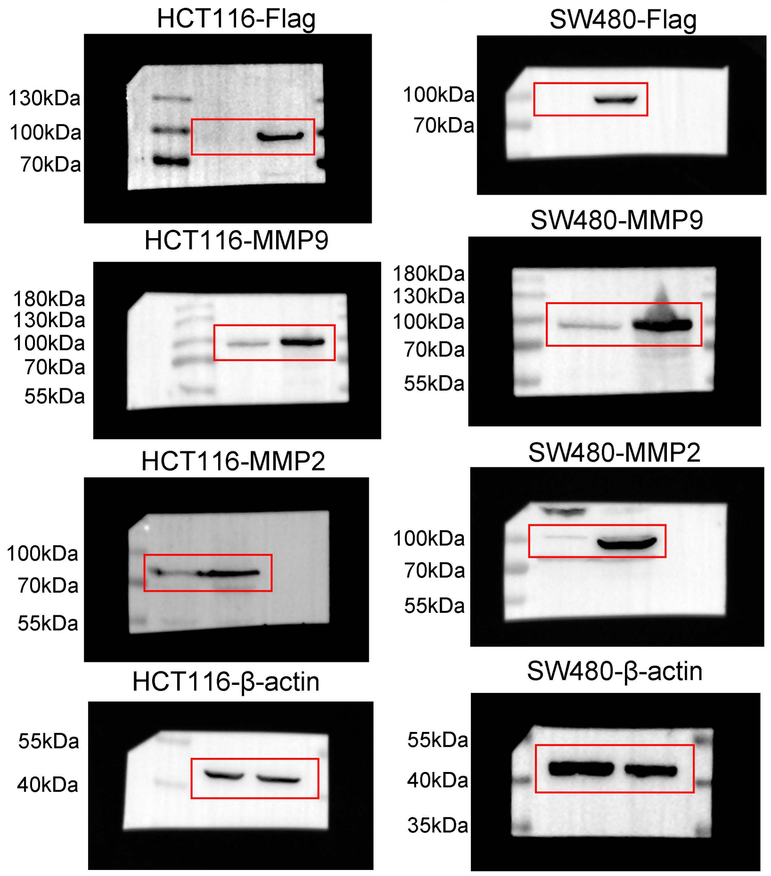

Figure-3L

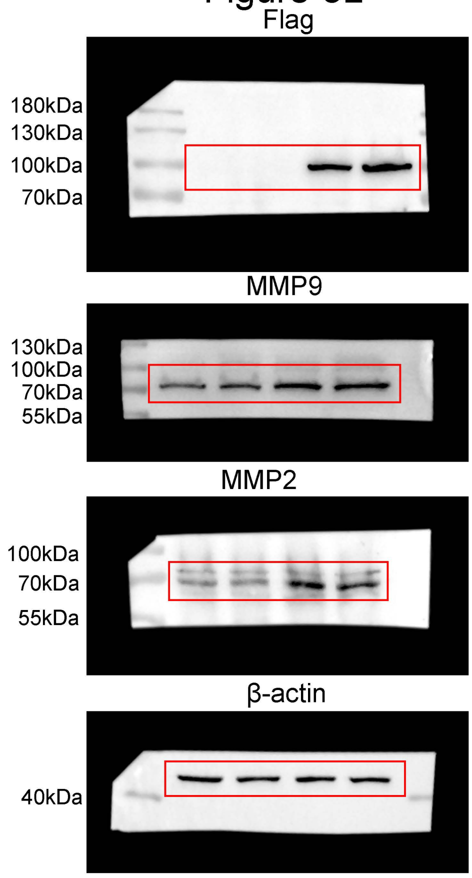

Figure-4F

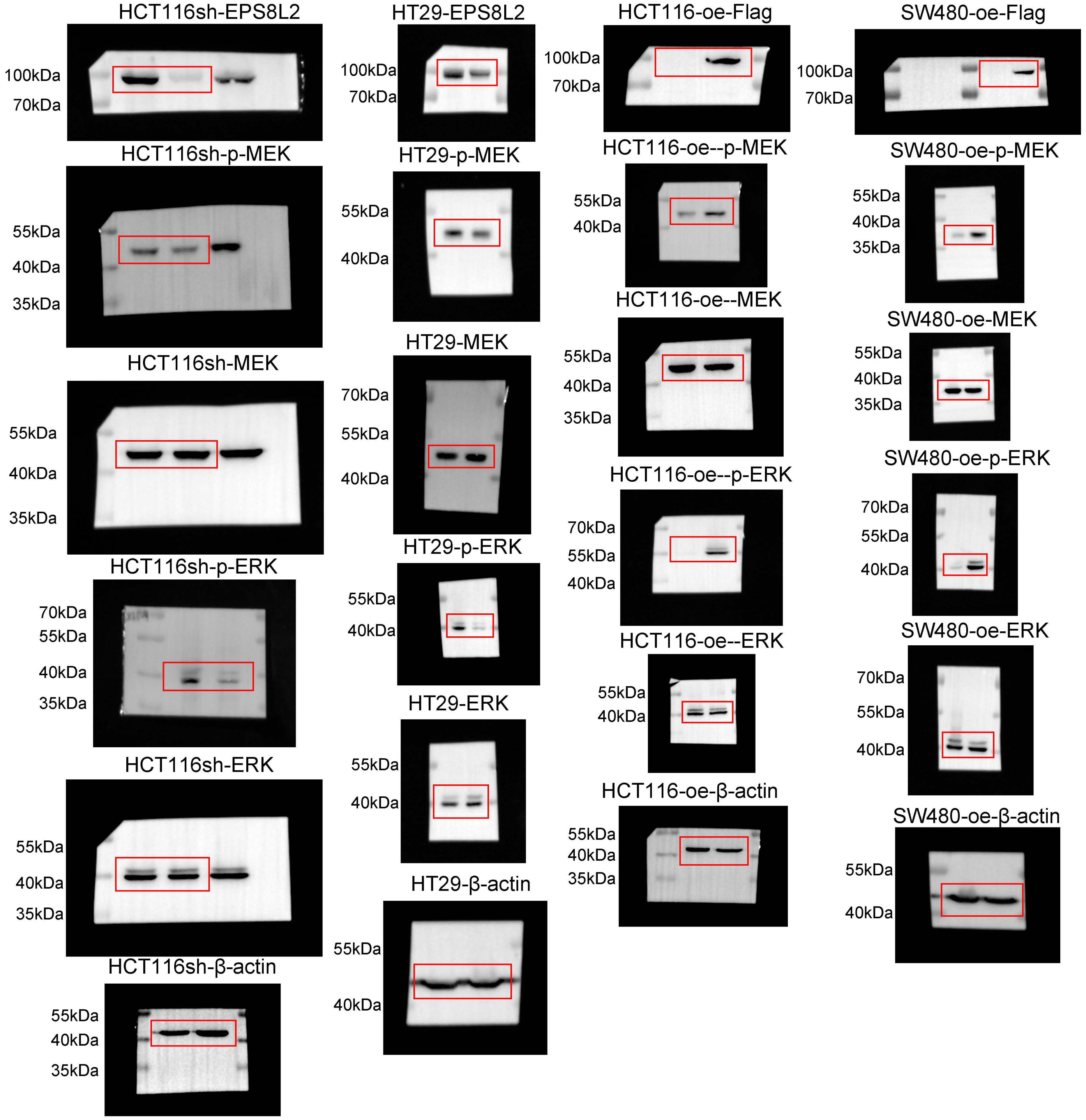

Figure-4G

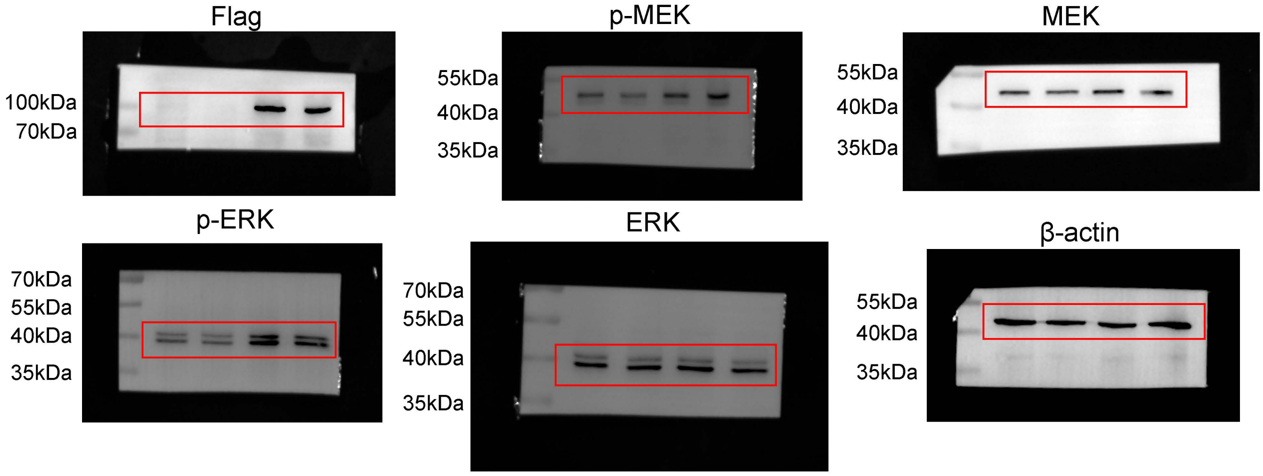

Figure-5C

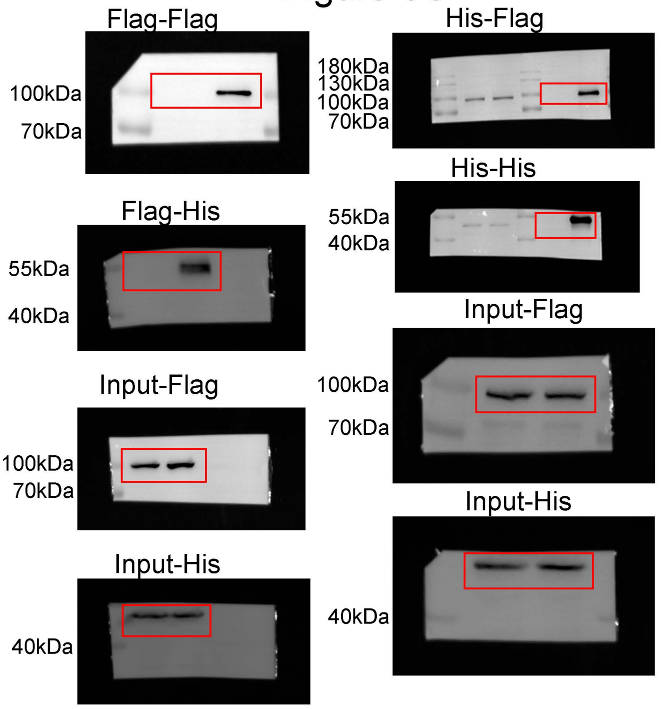

Figure-5D

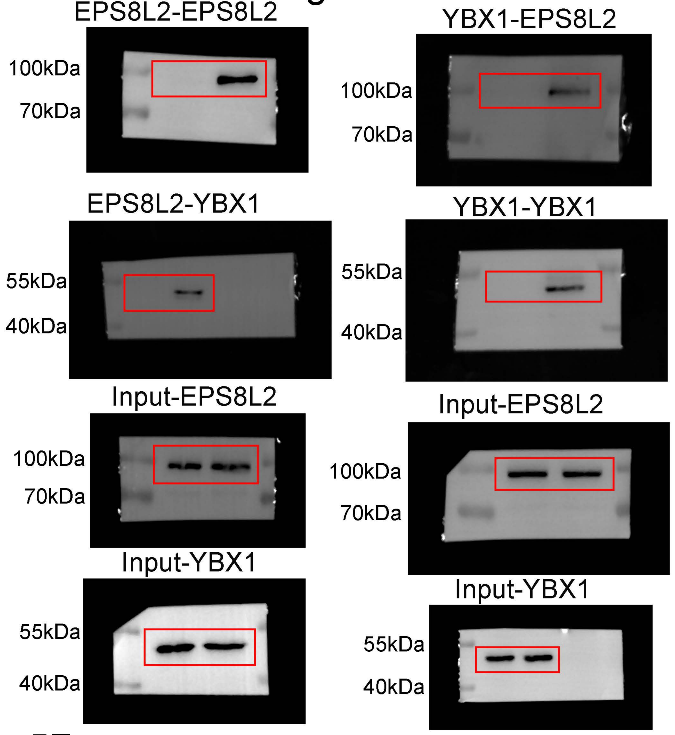

Figure-5F

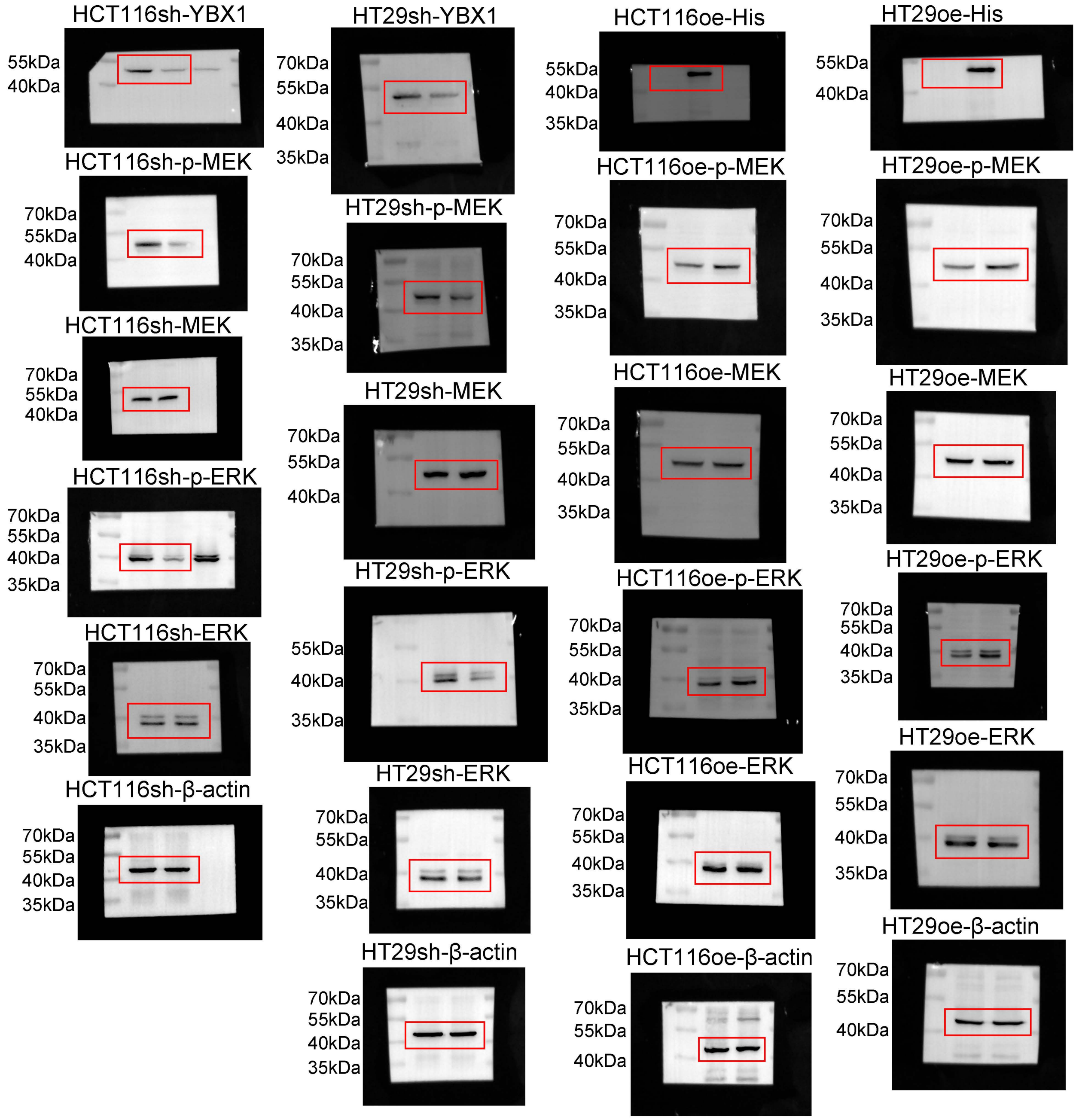

Figure-5G

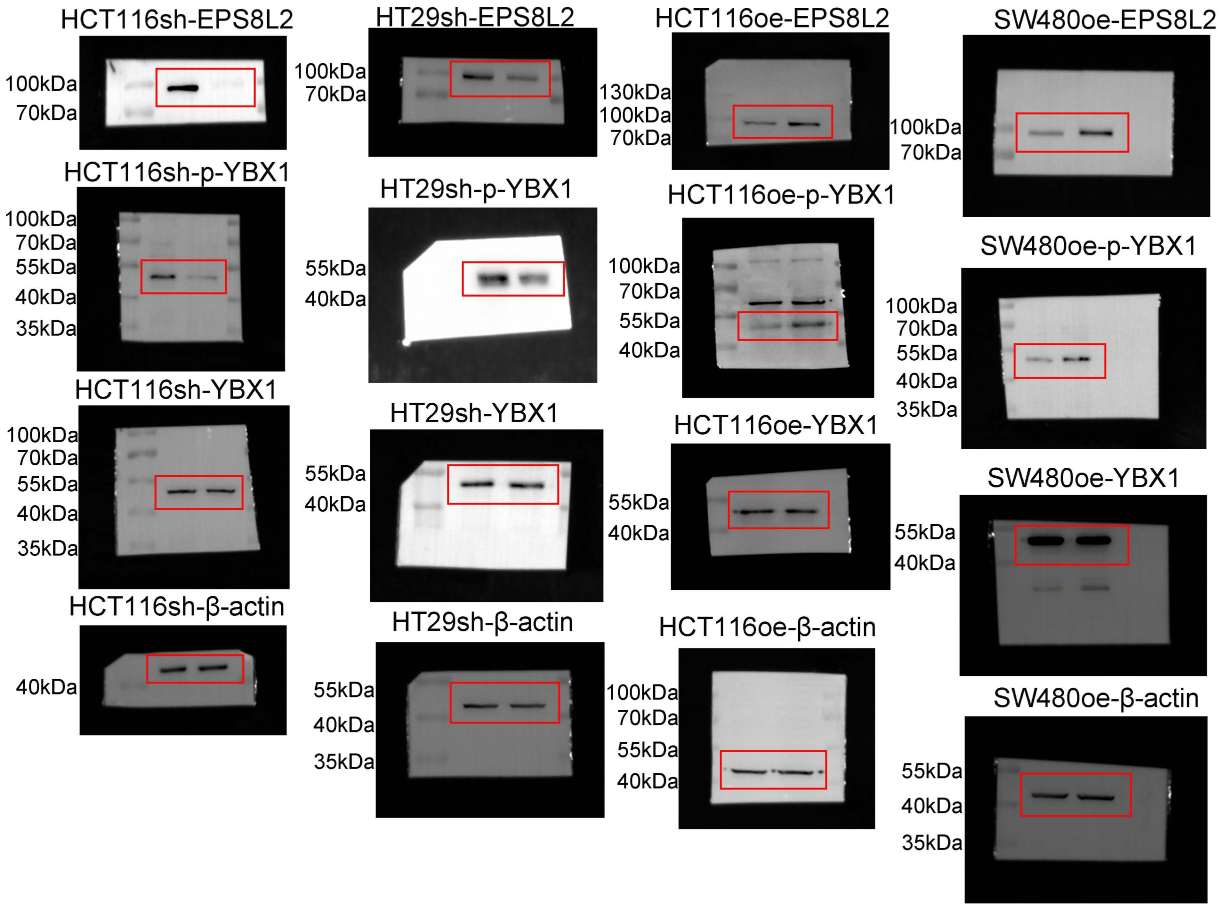

Figure-5H

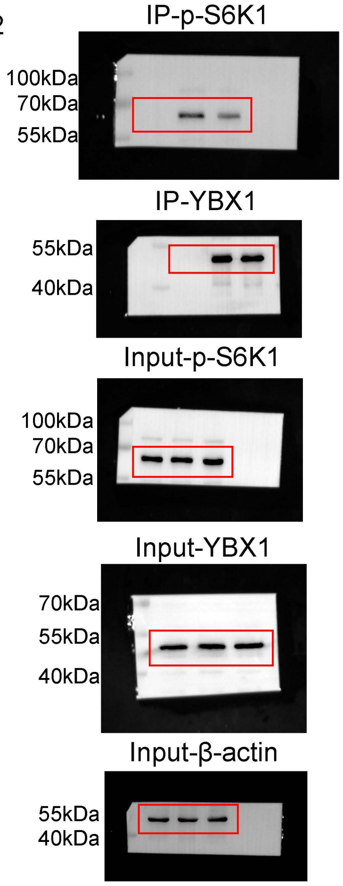

Figure-5I

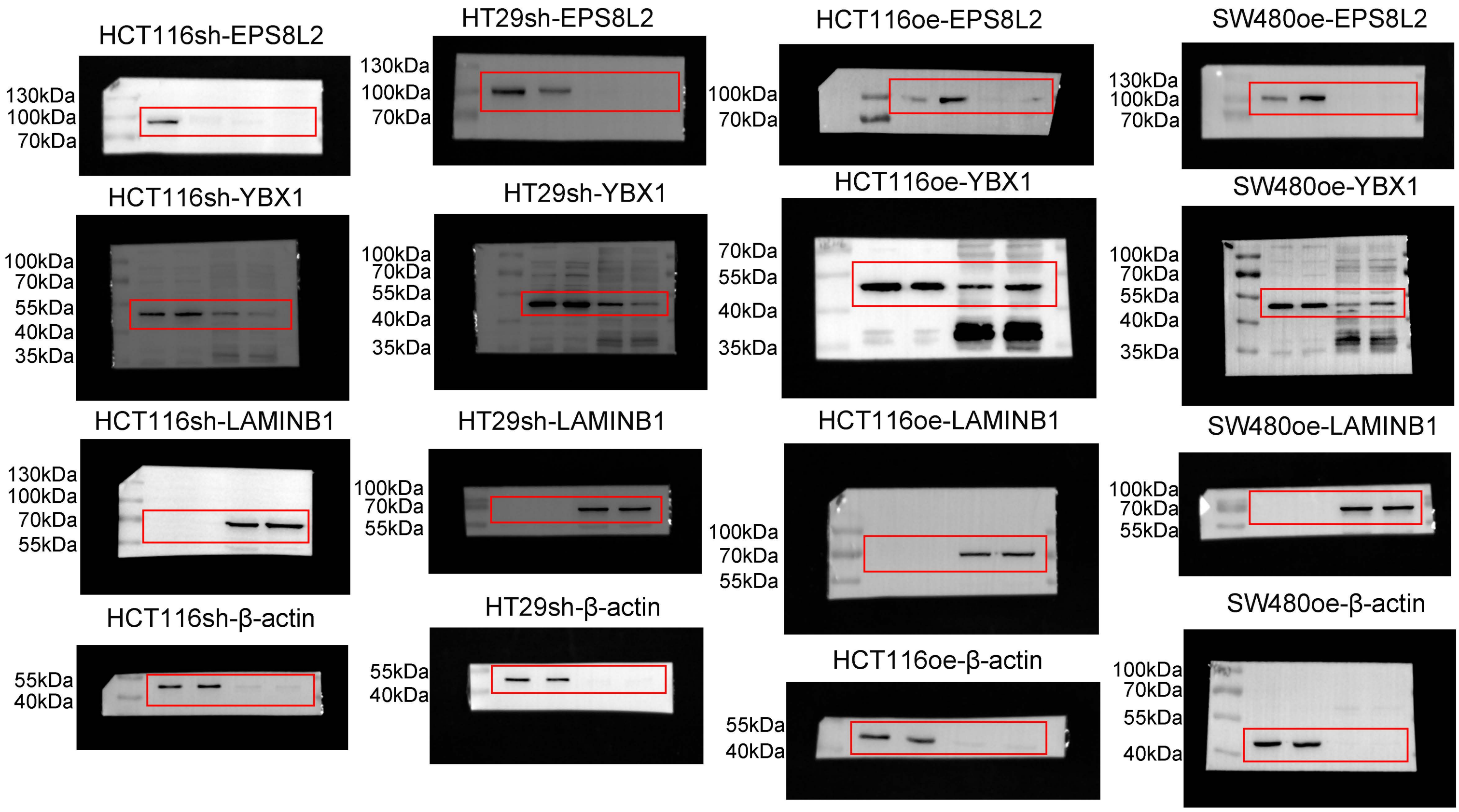

Figure-6I

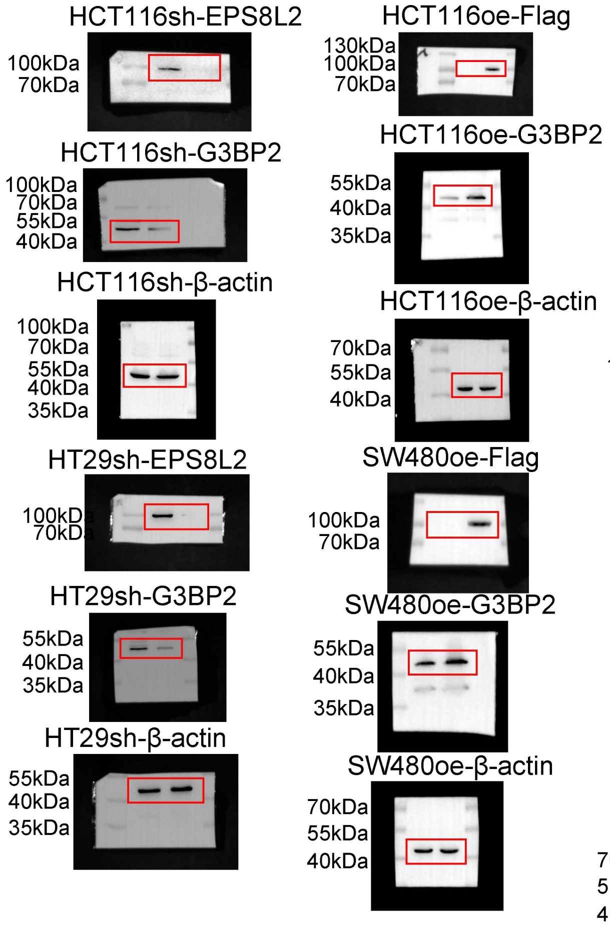

Figure-6J

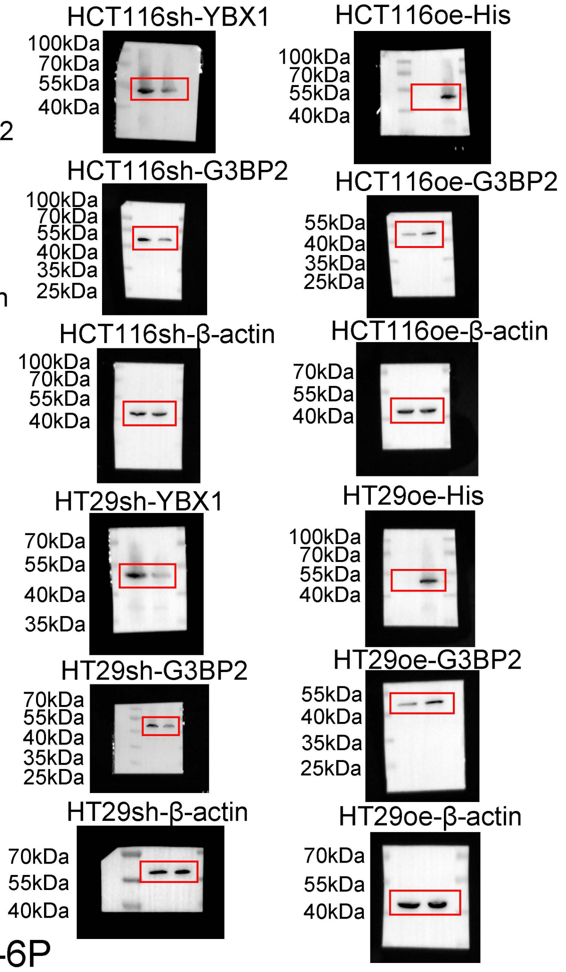

Figure-6P

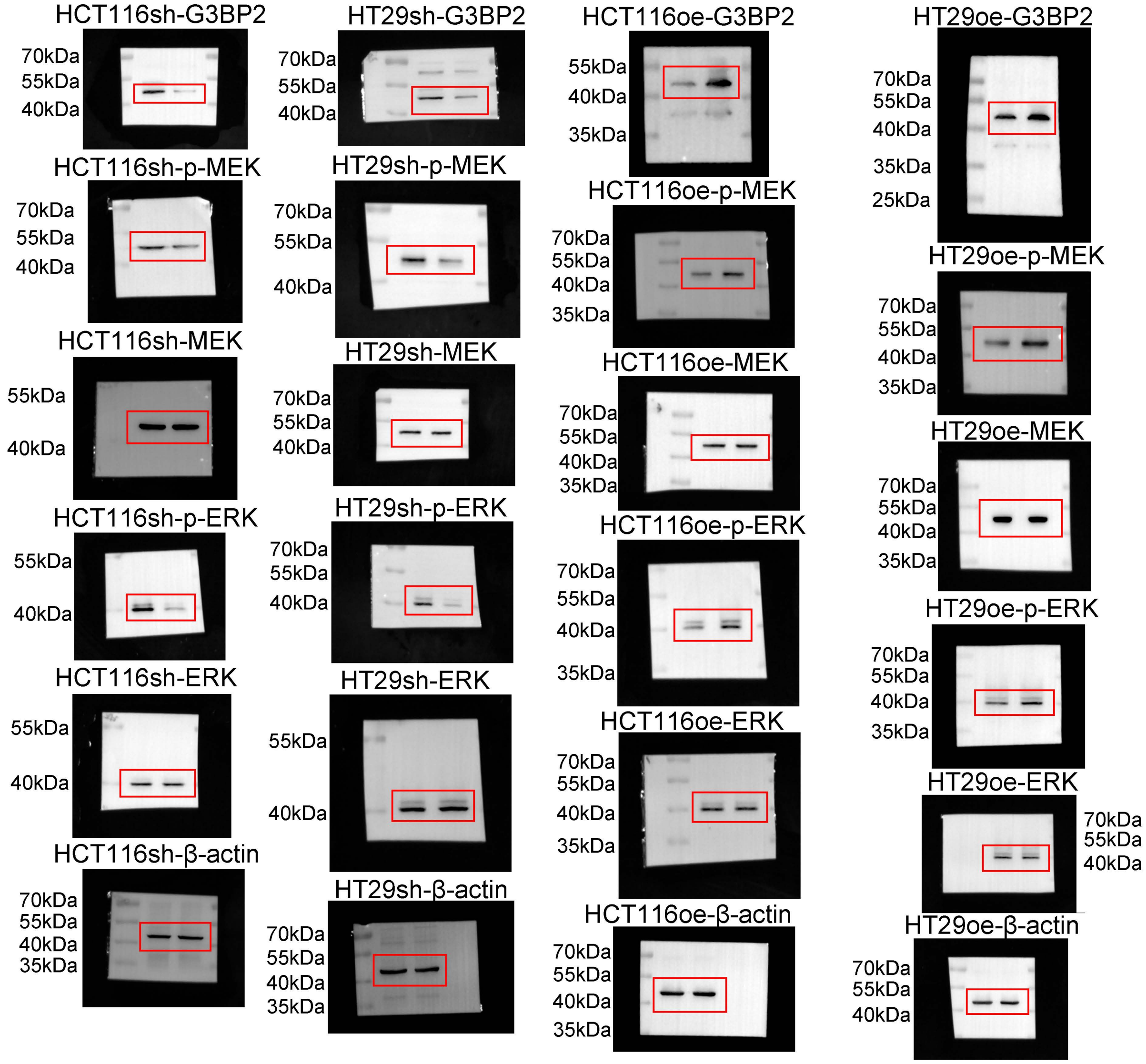

Figure-7I

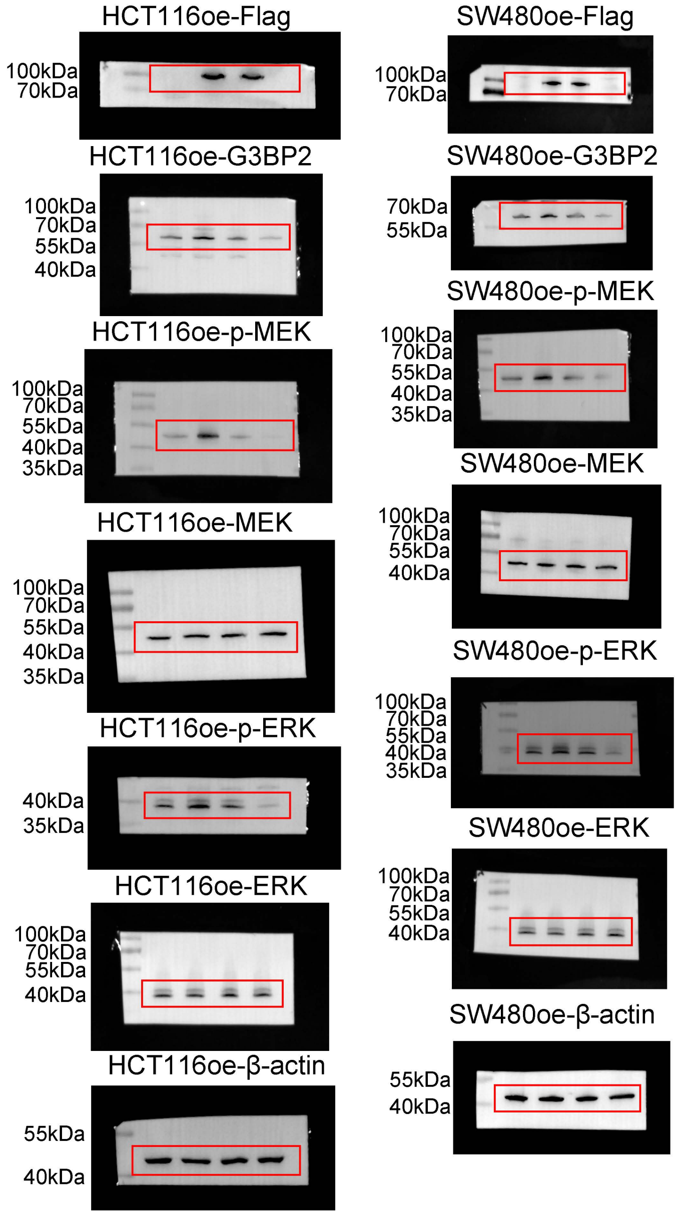

Figure-7J

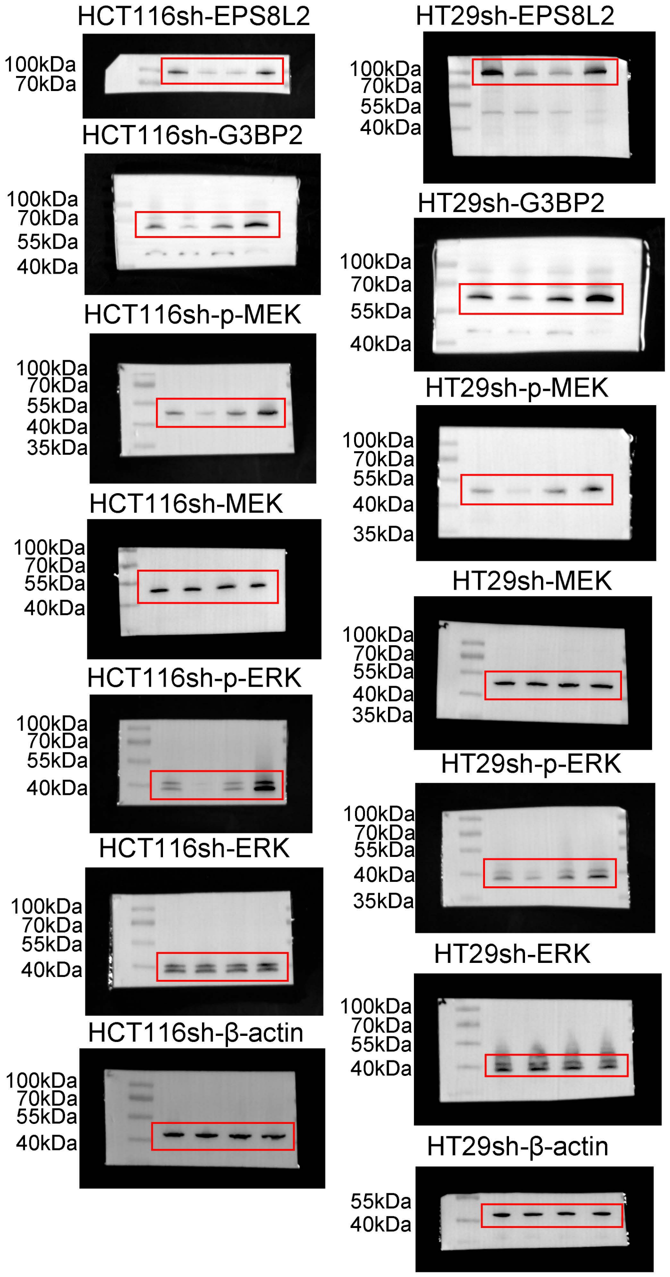

Figure-8B

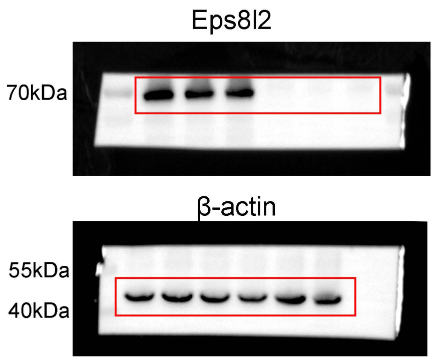

Figure-8J

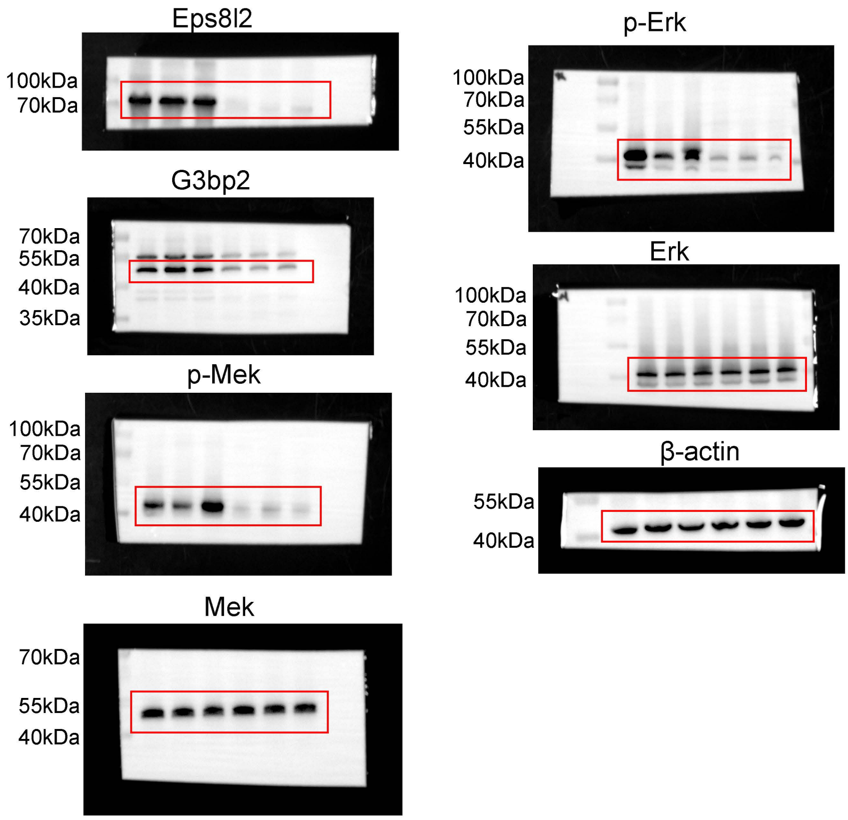

Figure-S2B

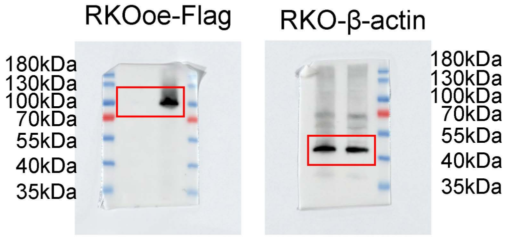

Figure-S4P

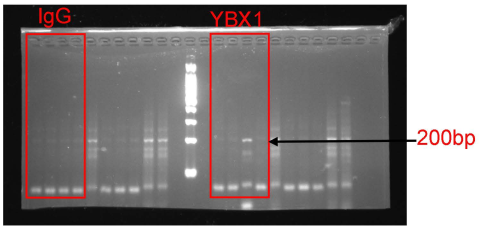

Supplement: Supplementary file 2 — uncropped western blots [file 41419_2025_7929_MOESM2_ESM.pdf]
